# Spatio-temporal expression pattern of Raffinose Synthase genes determine the levels of Raffinose Family Oligosaccharides in peanut (*Arachis hypogaea* L.) seed

**DOI:** 10.1038/s41598-023-27890-z

**Published:** 2023-01-16

**Authors:** Rajarshi Sanyal, Bhubaneswar Pradhan, Danish Md. Jawed, Kishor U. Tribhuvan, Anil Dahuja, Madan Kumar, Narendra Kumar, Gyan P. Mishra, Chet Ram, Mahesh Kumar Mahatma, Binay K. Singh, Satendra K. Mangrauthia, Anil K. Singh, T. R. Sharma, Arunava Pattanayak, Sujit K. Bishi

**Affiliations:** 1 School of Genomics and Molecular Breeding, ICAR–Indian Institute of Agricultural Biotechnology, Garhkhatanga, Ranchi, 834003 India; 2 Division of Biochemistry, ICAR-Indian Agricultural Research Institute, New Delhi, 110012 India; 3 Regional Research Station, ICAR-Directorate of Groundnut Research, Bikaner, 334006 India; 4 Division of Genetics, ICAR-Indian Agricultural Research Institute, New Delhi, 110012 India; 5 ICAR-Central Institute for Arid Horticulture, Beechwal, Bikaner, 334006 India; 6 ICAR-National Research Centre On Seed Spices, Beawar Road, Ajmer, 305206 India; 7 ICAR-Indian Institute of Rice Research, Hyderabad, 500030 India; 8 ICAR-National Institute of Plant Biotechnology, New Delhi, 110012 India; 9 Present Address: Department of Agricultural Biotechnology, Faculty Centre for Integrated Rural Development and Management, Ramakrishna Mission Vivekananda Educational and Research Institute, Kolkata, 700103 India; 10 Present Address: Career Point University, Alaniya, Kota, 325003 India

**Keywords:** Computational biology and bioinformatics, Molecular biology, Plant sciences

## Abstract

Raffinose family oligosaccharides (RFOs) are known to have important physiological functions in plants. However, the presence of RFOs in legumes causes flatulence, hence are considered antinutrients. To reduce the RFOs content to a desirable limit without compromising normal plant development and functioning, the identification of important regulatory genes associated with the biosynthetic pathway is a prerequisite. In the present study, through comparative RNA sequencing in contrasting genotypes for seed RFOs content at different seed maturity stages, differentially expressed genes (DEGs) associated with the pathway were identified. The DEGs exhibited spatio-temporal expression patterns with high RFOs variety showing early induction of RFOs biosynthetic genes and low RFOs variety showing a late expression at seed maturity. Selective and seed-specific differential expression of raffinose synthase genes (*AhRS14* and *AhRS6*) suggested their regulatory role in RFOs accumulation in peanut seeds, thereby serving as promising targets in low RFOs peanut breeding programs. Despite stachyose being the major seed RFOs fraction, differential expression of raffinose synthase genes indicated the complex metabolic regulation of this pathway. The transcriptomic resource and the genes identified in this study could be studied further to develop low RFOs varieties, thus improving the overall nutritional quality of peanuts.

## Introduction

Peanut (*Arachis hypogaea *L.) is a rich source of protein, calories, essential fatty acids, vitamins (mainly tocopherol and niacin), and minerals (like K, Ca, Fe, Zn, Mn, and Na). It is grown in more than 100 countries, with India and China ranking first in acreage and production, respectively, and has a global annual production of nearly 53.6 million tons^1^. Peanuts can fulfil the recommended daily protein requirement to the tune of 46%. Biologically active compounds like resveratrol, flavonoids, phytosterols and arginine make it even more attractive from a nutritional viewpoint. The World Health Organization (WHO) encourages the consumption of groundnut-based “ready-to-use therapeutic foods” (RUTF) to promote malnutrition-free communities^2^. Despite being a cheap, nutrient-dense food source, per-capita consumption of peanuts is quite low^2^. Peanut allergy, caused by some kernel proteins (like arachin and conarachin), saponins and tannins, food poisoning due to fungal (*Aspergillus flavus)* contamination releasing aflatoxin, and flatulence caused by Raffinose Family Oligosaccharides (RFOs) are the major factors limiting peanut consumption worldwide.

RFOs representing raffinose, stachyose and verbascose, are ubiquitous non-reducing plant carbohydrates formed by α-1,6-galactosyl extensions of sucrose. They are used as an alternate source of carbohydrates and are commonly found in seeds, especially in the family Leguminosae^3^, where they are considered the immediate energy source during germination^4^. RFOs are also reported to be present in leaves and tubers^5^. Two pathways have been reported for RFOs biosynthesis in plants; galactinol-dependent and independent pathways. In the dependent pathway, UDP galactose produced from galactose, and Myoinositol produced from glucose, form the initial precursors for RFOs biosynthesis^6^. Myoinositol serves as a common precursor for phytate and raffinose pathway; while galactinol formed via galactinol synthase (*GolS*) is used for RFOs generation and serves as the galactosyl donor to the members of this pathway. Sucrose and galactinol produce raffinose with the help of raffinose synthase and subsequently, stachyose and verbascose are formed by the stachyose synthase (*SS*) and verbascose synthase (*VS*), respectively. A galactinol-independent pathway also functions in certain plants^7^ where the enzyme galactan: galactan galactosyltransferase (*GGT*) catalyzes the galactosyl moiety transfer from one RFOs to another^8^. The fact that the galactinol-independent pathway has been reported to occur only in leaves, this pathway is probably absent in seeds^9^. RFOs are characterized as compatible solutes involved in stress tolerance mechanisms, despite evidence suggesting that they act as antioxidants. These sugars participate in carbon partitioning strategies and may have stress-induced signaling roles^10^. Despite the physiological and growth benefits of RFOs for plants, these are difficult to digest in humans and other monogastric animals due to the absence of the required enzymes that leads to abdominal discomfort and flatulence^11^. Further, the food containing higher RFOs take shorter time to pass through digestive tract, reducing absorption of other nutrients from feed. Together, these limit the consumption of crops with higher RFOs content.

Genes synthesizing RFOs shows tissue specific expression in response to various stresses. Seed specific *GolS* expression in response to desiccation^12^ and leaf specific expression during drought or heat stress^13^ highlights the importance of this gene in regulating the RFOs pathway. Several genes from *GolS* family have been known to play a regulatory role in Arabidopsis^14^, and reports on few *GolS* genes are also available in maize (*ZmGolS1/2/3*)^15^, rice (*OsGolS1/2*)^16^, chickpea (*CaGolS1/2*)^17^, mustard (*BnGolS1*)^18^, wheat (*TaGolS1/2*)^19^ and cucumber (*CsGolS1*)^20^. Molecular characterization of few raffinose synthases (*RS*) has been done in pea (*PsRS*)^8^, rice (*OsRS5*)^21^, soybean(*GmRS2*)^22^, maize (*ZmRS8*)^23^, cucumber (*CsRS*)^20^, lentil (*LcRS*)^24^ and grapes (*VviRS5*)^25^. Mutations as well as silencing of a raffinose synthase gene (*GmRS2*) of soybean generated phenotypes with low raffinose content^26,27^. Similar studies on common bean (*Phaseolus vulgaris*) identified seed specific galactinol synthase (*PvGolS1*) and raffinose synthase (*PvRS2*) genes, making them interesting candidates to knock out towards nutritional quality^28^. A genome-wide analysis recently identified *AdGolS3* as a candidate for drought tolerance in wild type peanut genotypes^29^. Despite RFOs being the major soluble sugar, after sucrose, in legumes^30^, there exists little information about the genes metabolizing RFOs in Fabaceae species. Genomic resources for peanut have been increasing for the last few years and the availability of complete genome sequences (https://peanutbase.org/) have enabled researchers to gain new perspectives.

In the recent years, RFOs have been projected as a prebiotic promoting certain types of beneficial gut microbes in vitro^31^. However, their anti-nutritional effects in humans and animals still outweigh such beneficial properties, especially in crops like peanut, which is consumed both uncooked and cooked and as raw cakes for animal feed after oil extraction. Unlike other legumes, cooked or sprouted peanuts, which reduce inherent RFOs levels^32^, are not preferred by the majority of consumers. Moreover, it becomes necessary to reduce the RFOs level in the kernels so that the abiotic stress tolerance which is majorly influenced by leaf RFOs levels^28^, get minimum hindrance. Therefore, controlled production of RFOs in peanuts through genetic manipulations appears to be an attractive option to produce varieties where the RFOs content suits the requirements of both plant and the eater. With this backdrop, this study aimed at identifying the candidate genes imparting differential RFOs accumulation within and between peanut botanical groups, using comparative transcriptomic approach.

## Results

### Content and Composition of RFOs and their temporal variation in seed

**Figure 1:**
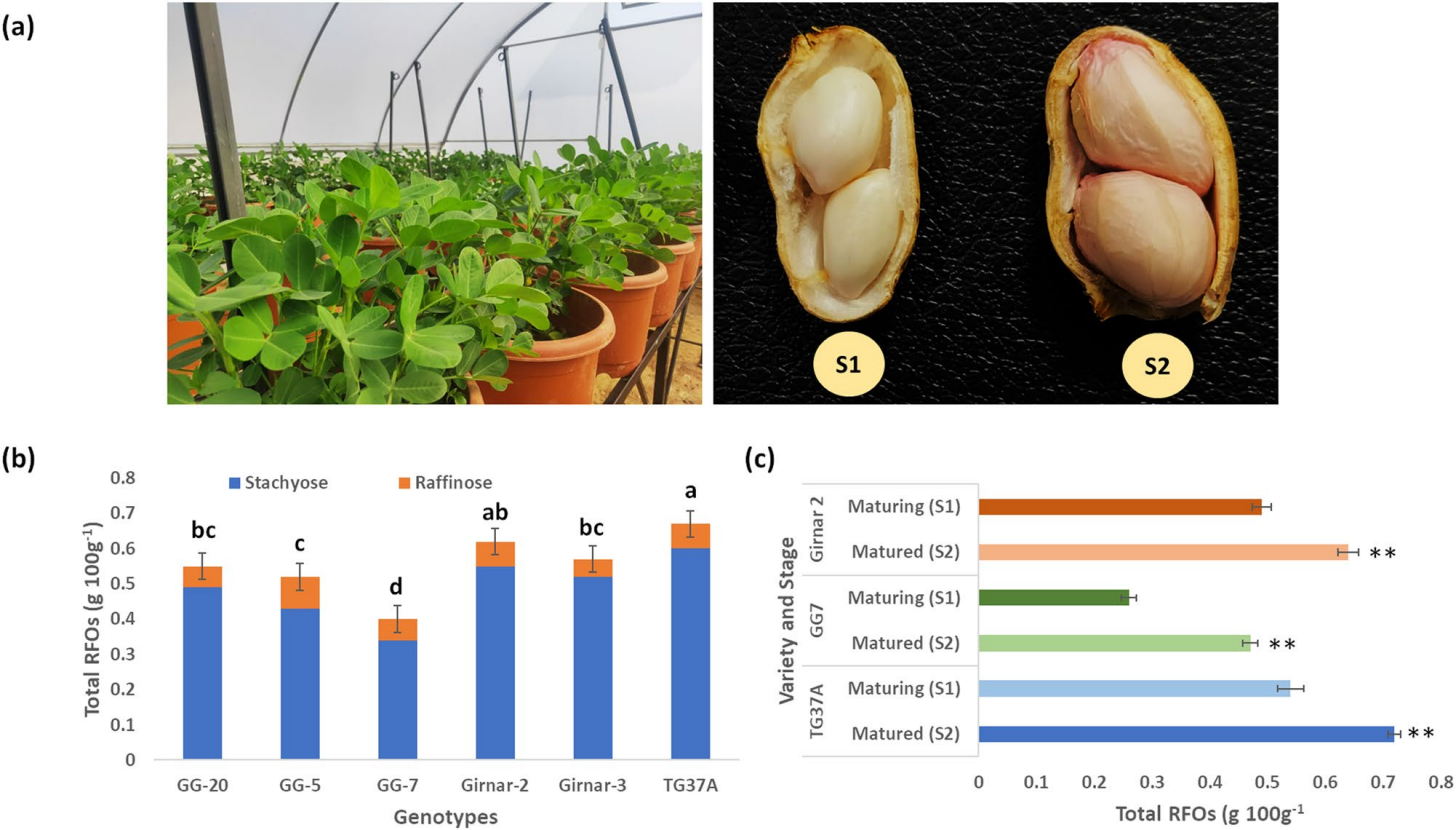
**(a)** Growing conditions in the greenhouse (left) and the seed stages (right) at which the samples were collected. S1 represents maturing stage and S2 represents matured stage **(b)** Content of RFOs (g/100 g) in the dry seeds of six peanut varieties. **(c)** RFOs content (g/100 g) in the maturing stage (S1) and matured stage (S2) of seeds in contrast peanut varieties. All values are mean of three replications. Mean values with different letters or asterisks represent the statistical difference (p-value ≤ 0.05).

In order to confirm the previously reported variations in RFOs content and composition, six peanut varieties, representing both Spanish (Girnar 3, GG-5, GG-7, and TG-37A) and Virginia (Girnar 2, and GG-20) groups, growing under greenhouse conditions (Fig. 1a) were analysed. Total RFOs content varied from 0.40% in GG7 to 0.67% in TG37A with a mean of 0.56% on dry weight basis (Fig. 1b). In the Virginia group, Girnar 2 showed a comparatively higher RFOs content (0.62%) than GG20 (0.55%). Based on the significant difference in RFOs content, contrasting varieties within and between groups (TG37A, GG7 and Girnar 2) were selected for further study. Raffinose content showed almost similar trend in all the varieties, with a mean value of 0.7 mg/g (Supplementary Table S5 online). Stachyose content significantly varied among the varieties (3.4–6.0 mg/g) with mean value of 5.0 mg/g. GG7 contained the lowest stachyose content (3.4 mg/g) while TG37A contained the highest content (6.0 mg/g). The mean sucrose content in the tested varieties was 6.23 g/100 g and ranged from 5.27 to 7.08 g/100 g. Significant variations in glucose content was also recorded (0.001 to 0.008 g/100 g) among the studied varieties. Inositol content ranged from 0.04 to 0.09 g/100 g with an average of 0.06 g/100 g. A positive correlation was observed between sucrose and raffinose (r = 0.64; *p*-value = 0.004) while stachyose was strongly correlated to total RFOs (r = 0.99; *p*-value = 0.000) and formed the major component (approximately 90%) of seed RFOs.

Enzyme based assessment of RFOs accumulation in the different stages of seed (Fig. 1a) revealed a significant variation in the RFOs content between the stages in the contrasting varieties (Fig. 1c). In TG37A, the RFOs content was higher in S2 (0.72%) when compared to S1 (0.54%). GG7 also showed a higher RFOs content in S2 (0.47%) when compared to S1 (0.26%). In TG37A and Girnar 2, nearly 1.3 fold increase in total RFOs content was recorded at S2 when compared to S1. Further, an increase of about 1.8 fold was observed in GG7 (Supplementary Table S6 online). Invariably, the sucrose content got depleted with maturity and recorded nearly 40.5%, 38.2%, and 36.2% reduction in TG37A, Girnar 2, and GG7, respectively. No significant variation in glucose content was observed in GG7, but TG37A and Girnar 2 showed a slight reduction as the seed reached its maturity. Thus, the biochemical analysis confirmed an increasing trend in RFOs accumulation towards seed maturity, with a higher RFOs content in TG37A and Girnar 2 (high RFOs containing varieties); while a higher fold change in GG7 (a low RFOs variety).

### RNA sequencing and Reference based mapping statistics

To explore the reasons for this differential accumulation of RFOs in contrasting peanut varieties, RNA seq was performed which resulted in nearly 42 million raw reads per sample, of which 98.4% were clean reads. High Q30 value (about 98%) and Read Alignment (> 99%) was obtained for all the samples. On an average, 61,937, 57,159, and 59,231 genes with at least 1 mapped read were found in TG37A, GG7, and Girnar 2, respectively (Table 1).

**Table 1:** Summary of RNA-seq data of three peanut seed samples collected at two stages of maturity.

Sample name		Parameters					
		Raw reads	Clean reads	Read alignment (%)	GC content (%)	Q30 (%)	Unigenes (No) with FPKM ≥ 1
TG37A_S1	R1	25,375,298	25,012,580	99.61	46	98.23	57,123
	R2	17,529,282	17,302,971	99.77	46	98.24	58,233
	R3	25,367,741	24,985,543	99.48	43	98.14	65,157
TG37A_S2	R1	22,454,235	22,178,624	99.12	47	98.42	62,748
	R2	14,557,272	14,384,373	95.79	52	98.79	63,203
	R3	18,147,617	17,807,553	99.57	45	97.95	57,942
GG7_S1	R1	19,582,678	19,235,444	99.93	46	97.71	59,043
	R2	25,901,692	25,418,620	99.73	47	97.66	56,034
	R3	25,724,893	25,321,295	99.54	47	98.17	61,205
GG7_S2	R1	26,414,843	25,979,311	99.78	47	98.02	55,494
	R2	14,461,718	14,215,239	99.89	47	98.09	55,320
	R3	21,690,814	21,322,986	99.87	47	98.10	55,855
Girnar2_S1	R1	19,658,240	19,362,886	98.91	49	98.14	67,561
	R2	23,035,959	22,665,409	99.84	47	97.98	56,714
	R3	21,660,161	21,282,869	99.80	45	97.96	59,192
Girnar2_S2	R1	16,360,833	16,094,047	99.63	45	98.02	58,586
	R2	22,712,357	22,335,104	99.79	47	98.03	54,893
	R3	17,465,233	17,154,113	99.72	46	97.88	58,442
Total	378,100,866	372,058,967					

Seed maturity stages are denoted by S1 (maturing stage) and S2 (matured stage). R1, R2, R3 are the three biological replicates of each sample. Q30 = Quality Score 30, FPKM = Fragments Per Kilobase of transcript per Million mapped reads.

On an average 99.43% of the reads were aligned onto the reference peanut genome (https://peanutbase.org/data/v2/Arachis/hypogaea/genomes/Tifrunner.gnm1.KYV3/), while uniquely mapped reads were 83.93%. In TG37A_S1, 1,10,76,166 reads were obtained, of which 99.62% were mapped, and 88.29% were uniquely mapped. TG37A_S2 had 77,13,500 reads, of which 98.16% were mapped, and 87.90% were uniquely mapped. On an average, 77,22,841 reads were obtained in GG7_S1 and of which 99.73% were mapped, and 81.7% were uniquely mapped. For GG7_S2 sample, 94,38,066 reads were obtained, 99.85% of which were mapped, and 78.69% were uniquely mapped. In Girnar 2, 95,87,507 reads were obtained in S1 while for S2, 1,04,39,017 reads were obtained. Of these, 99.52% and 99.71% of the reads were mapped in S1 and S2, respectively. Further, 81.74% (at S1) and 85.25% (at S2) were uniquely mapped while 0.48% (at S1) and 0.29% (at S2) of reads remained unmapped.

### Gene expression analysis using RNA-Seq data

**Figure 2:**
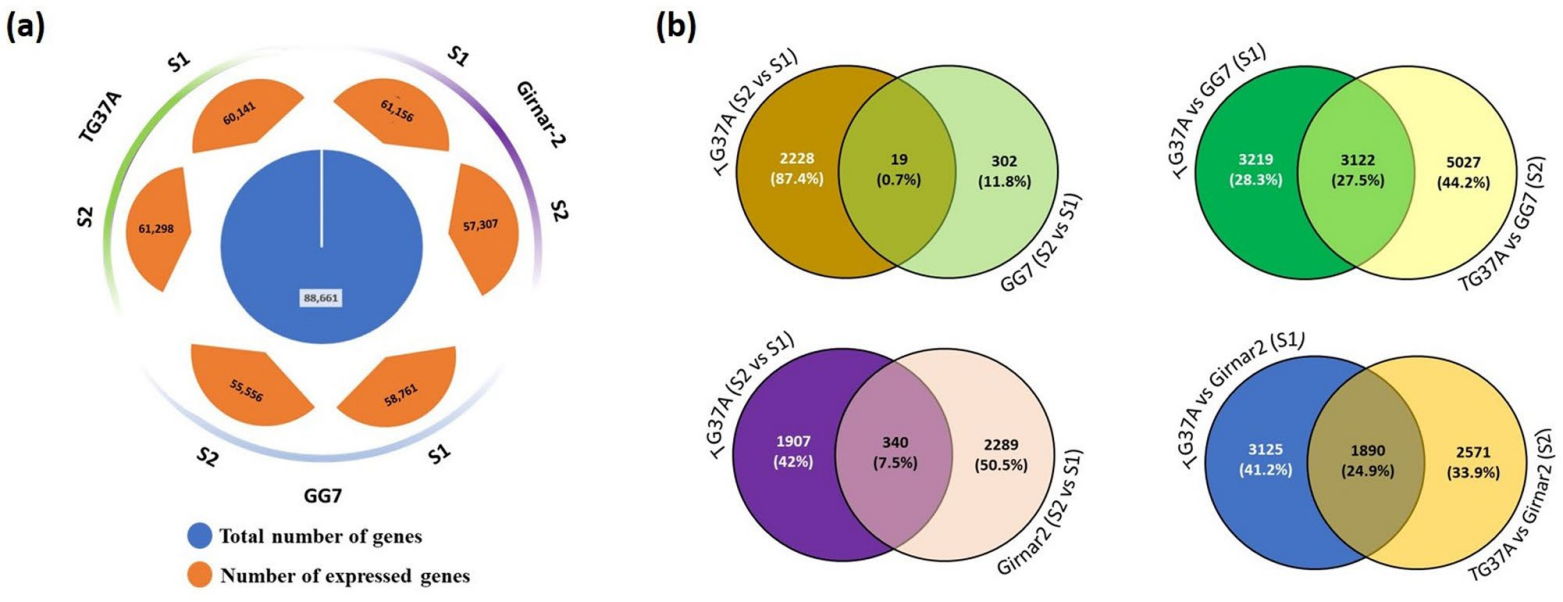
Diagrammatic representation of the number of Differentially Expressed Genes (DEGs) in different combinations of peanut seed sample. **(a)** Genes with at least one mapped read have been considered. The total number of genes has been represented in a blue circle while the number of expressed genes (orange) in TG37A (green), Girnar 2 (purple), and GG7 (light blue) at maturing seed (S1) stage and matured seed (S2) stage have been represented using different colors. **(b)** The figure has been partially designed using venny 2.1 webtool (https://bioinfogp.cnb.csic.es/tools/venny/index.html). Seed maturity stages has been denoted by S1 (maturing seed stage) and S2 (matured seed stage). Combinations of two samples have been represented with versus (vs).

Reference based mapping statistics generated 88,661 genes, of which, 60,171 (68%) and 62,902 (71%) genes were expressed in TG37A maturing (S1) and matured (S2) seed stage, respectively. Girnar 2 exhibited expression of 61,156 (69%) genes at S1 and 57,307 (65%) genes at S2 while GG7 showed expression of 58,761 (66%) genes at S1 and 55,556 (63%) genes at S2 (Fig. 2a).

Differential Expression (DE) analysis showed significant expression of 3.7% of the tested genes (62,987) in GG7, of which, 26.8% were upregulated and 73.2% were downregulated at S2 over S1. Similarly, in TG37A, 3.6% of genes showed significant expression and of 2247 DEGs, 13.4% were upregulated and rest were downregulated. In Girnar 2, 4.2% of the genes showed upregulation (of 15.8% of expressed genes), while 84.2% showed downregulation. When contrasting varieties (TG37A and GG7) were analyzed at S1, 10% genes showed significant expression and of these, 42.4% were upregulated, while 57.6% were downregulated. When analyzed at S2, 13% of the genes were significantly expressed and of these, 63.8% were downregulated, while rest showed upregulation. On comparing TG37A and Girnar 2, 7% and 8% of tested genes (62,987) showed significant expression at S1 and S2 respectively. Almost similar trend was observed in both the seed stages with upregulation of 35.4% genes (at S1) and 31% genes (at S2) and downregulation of 64.6% genes (at S1) and 69% genes (at S2). (Supplementary Table S7 online). The Venn diagram revealed 19 common genes (0.7%) at S2 in both TG37A and GG7 while 302 unique genes (11.8%) in GG7, and 2228 unique genes (87.4%) in TG37A when compared to S1. The expression of 3122 genes (27.5%) was found common at both S1 and S2 stages while 3219 genes (28.3%) and 5027 genes (44.2%) were found uniquely expressed at S1 and S2, respectively. Comparison between high RFOs varieties belonging to Spanish (TG37A) and Virginia (Girnar 2) groups, revealed the expression of 1890 genes (24.9%) as common at both seed stages with unique expression of 3125 genes (41.2%) at S1 and 2571 genes (33.9%) at S2 stages (Fig. 2b). Moreover, of 4536 genes, 1907 (42%) were specifically expressed in TG37A, 2289 (50.5%) were specific for Girnar 2 and 340 genes (7.5%) were common. To summarize, when the contrast varieties were compared at S1 and S2 stage, significant number of genes played a common role in the seed developmental processes, irrespective of the group (Spanish or Virginia) but when compared within the seed stages of individual variety, most of the genes uniquely contributed to the developmental change, suggesting a differential behaviour.

### Identification of differentially expressed genes (DEGs) during different stages of maturity in high and low RFOs varieties

**Figure 3:**
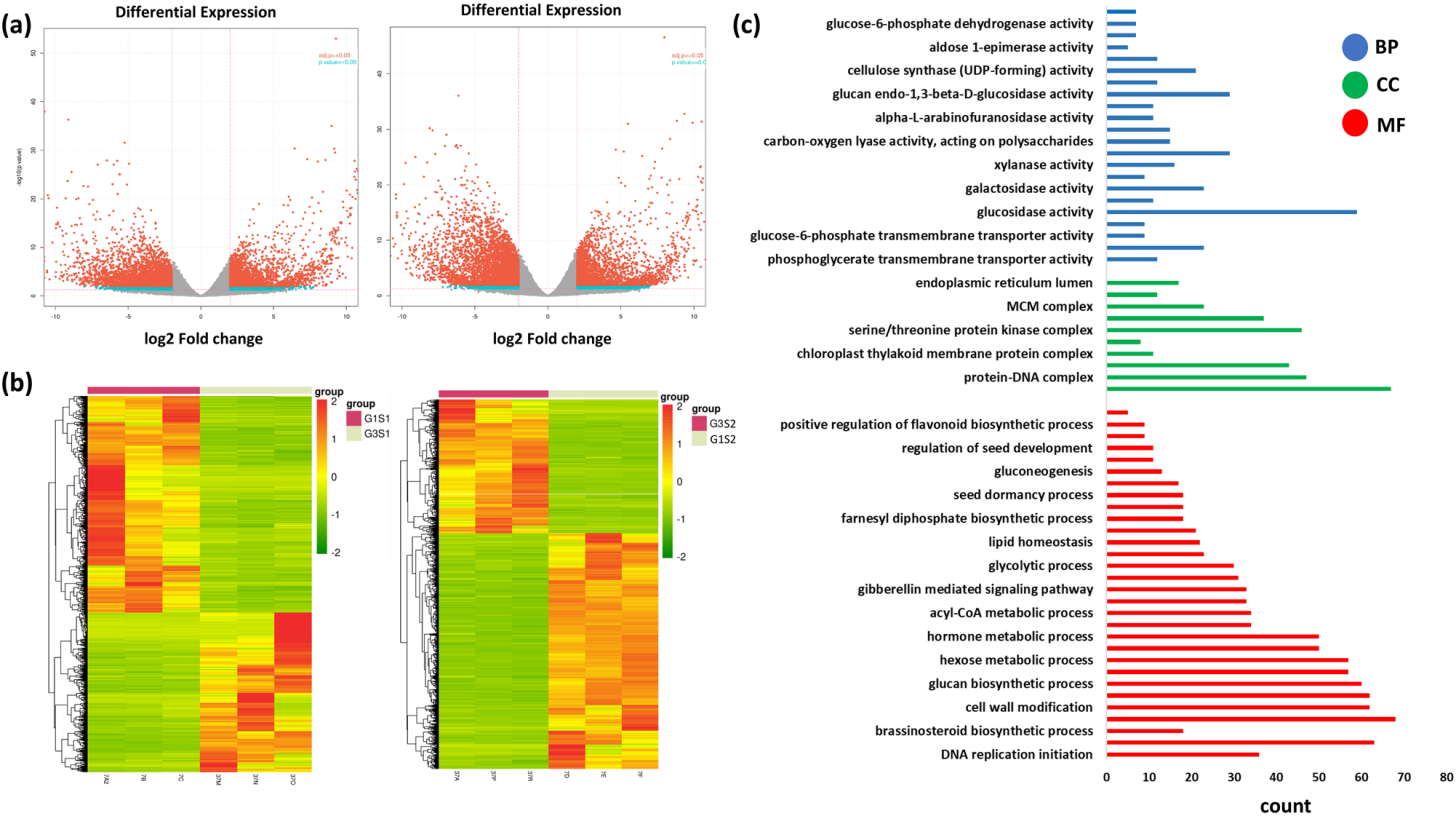
Representative expression profile and GO categorization of peanut seed transcriptome of the Spanish group varieties. **(a)** Volcano plot showing differential expression profile of genes. “Blue” indicates log2 fold change ≥ 2 and *p* value ≤ 0.05. “Red” indicates absolute log2 fold change ≥ 2 and adjusted *p* value ≤ 0.05. **(b)** Heatmap showing the DEGs in GG7 vs TG37A at S1 (left) and S2 (right). G1 = GG7 (red coloured cluster), G3 = TG37A (beige coloured cluster). Data is plotted with the *heatmap.2* function of R package *gplots* (version 3.1.1). **(c)** These GO terms are classified into three categories (BP: Biological Processes, CC: Cellular Component, MF: Molecular Function).

The identified DEGs from the contrasting varieties (at different stages) were blasted against the Uniprot-GOA (Gene Ontology Annotation) database and were categorized into altered metabolic processes like biological processes, cellular compartments, and molecular functions. A number of genes showed significant expression during maturing (S1) and matured (S2) stage of the seed (Fig. 3a) The DE of genes at two maturity stages among the contrast varieties (high and low RFOs), as shown in the Fig. 3b, displayed a striking difference in the expression of seed development related genes at S1 and S2 stage.

For biological processes (BP), cell cycle and cell wall modification were the major groups in the early stage of seed maturity whereas processes like pigment biosynthesis, hexose metabolism, and glucan biosynthesis played decisive roles during later stages (Fig. 3c). Anchored components of plasma membrane, photosystem, and protein DNA complex were the major cellular components associated with both the varieties, irrespective of the seed stage. While protein kinase complexes played a major role during early stage, vesicle membranes were predominant in the later stage of maturity. Vitamin binding, enzyme inhibitor activity, glucosidase activity were the top molecular functional groups in both seed stages but maturing stage was characterized by activities like carbon–oxygen lyase and transferase while matured stage was characterized by oxidoreductase activity and transmembrane signalling activity. Processes related to carbohydrate metabolism like glucose 6 phosphate dehydrogenase activity, cellulose synthase (UDP-forming), galactosidase, glucosidase, arabinofuranosidase, polysaccharide lyases were also found to be major contributors in the seed stages of the contrast varieties. The analysis also revealed alteration in the processes like lipid biosynthesis, flavonoid biosynthesis, brassinosteroid and gibberellin signaling especially during seed maturity in peanut (Fig. 3c).

### Identification of DEGs involved in RFOs biosynthesis in seeds

**Figure 4:**
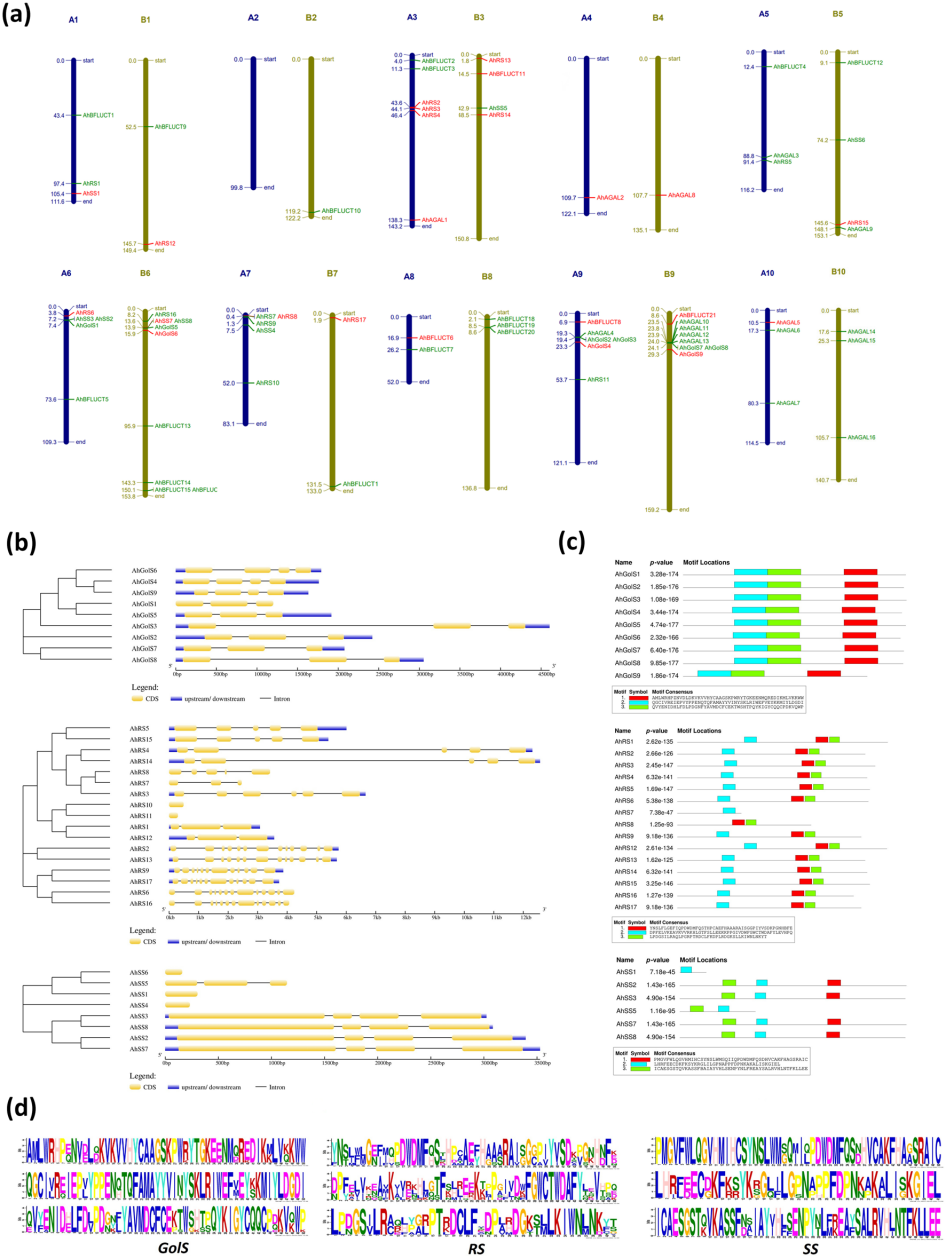
Characterization of RFOs biosynthetic genes in peanut using structural genomics tools. **(a)** The chromosome wise distribution of Raffinose Family Oligosaccharide genes mapped to the A. *hypogaea* genome. The scale used for gene location depiction was taken in mega bases (Mb) and drawn using MapChart software. The Differentially Expressed Genes (DEGs) are marked with red. **(b)** Phylogenetic tree and structures of genes encoding the galactinol synthase (*GolS*), raffinose synthase (*RS*) and stachyose synthase (*SS*) enzymes visualized using GSDS software. **(c)** The motif compositions of A. *hypogaea GolS, RS and SS* genes were identified using MEME analysis tool. Each color representing a specific motif. **(d)** Statistically over-represented sequence within the differential peaks discovered by MEME-ChIP in RFOs biosynthetic genes.

Alterations in carbohydrate metabolism and signalling processes intrigued us to identify the members of galactinol synthase (*GolS*), raffinose synthase (*RS*), and stachyose synthase (*SS*) genes from the RNA Seq data which seems regulating the RFOs biosynthesis in peanut seeds. Using bioinformatics pipeline, eight out of nine members of *GolS* family (*AhGolS1*, *AhGolS2*, *AhGolS4*, *AhGolS5*, *AhGolS6*, *AhGolS7*, *AhGolS8*, and *AhGolS9*) present in peanuts were found in the seeds. Of these, *AhGolS4*, *AhGolS6*, and *AhGolS9* showed significant DE. Of 17 *RS* family genes, 16 were identified in the seed but the highest FPKM was observed in *AhRS6* and *AhRS16*. Of 16 members, ten (*AhRS2*, *AhRS3*, *AhRS4*, *AhRS6*, *AhRS8*, *AhRS12*, *AhRS13*, *AhRS14*, *AhRS15*, *and AhRS17*) showed significant DE. Eight *SS* family genes were also identified in peanuts, and of these 06 were found in the studied transcriptomics data. Except for *AhSS4* and *AhSS5*, all were identified, but higher FPKM was found associated with *AhSS2* and *AhSS7* genes. However, only two members (*AhSS1* and *AhSS7*) showed significant expression. Besides the biosynthetic genes, four (*AhAGAL1, AhAGAL2, AhAGAL5, AhAGAL8*) alpha-galactosidase (*AGAL*) and four (*AhBFLUCT6, AhBFLUCT8, AhBFLUCT11, AhBFLUCT21*) beta fructofuranosidase (*BFLUCT*) genes were also found involved in RFOs catabolism (Fig. 4a).

### In silico characterization of the DEGs involved in RFOs biosynthesis in seeds

In order to characterize the identified members, we have used a strategically designed bioinformatics pipeline. The identified members from *GolS* family, which catalyses the galactinol formation from myoinositol and UDP-D-Galactose, are distributed on the chromosome A6/B6 and A9/B9. *AhGolS1* is the only member found on chromosome A6 while chromosome B6 was found harbouring *AhGolS5* and *AhGolS6* genes. Other members like *AhGolS2*, *AhGolS3*, and *AhGolS4* are present on chromosome A9, and *AhGolS7*, *AhGolS8* and *AhGolS9* are located on chromosome B9 (Fig. 4a). *AhGolS4* and *AhGolS9* show high sequence similarity and, both found containing 04 exons and 03 introns (Fig. 4b) and may be considered as homeologs on chromosome A6/B6. Another homeologous pair containing 03 exons and 04 introns are *AhGolS1* and *AhGolS5*, and are located on chromosome A9/B9.

Seventeen possible *RS* gene members have been predicted in *A. hypogaea* and the nomenclature of this gene in most of the databases was “galactinol-gucrose galactosyl transferase”. Raffinose synthase catalyzes the conversion of galactinol to raffinose by transferring a galactose unit to the sucrose backbone. Members of this family are widespread with maximum members on chromosome A7 (Fig. 4a). A high sequence similarity between *AhRS5* and *AhRS15* was recorded, as both possess five exons and four introns and are situated on chromosome A5 and B5, respectively (homeologs). *AhRS4* and *AhRS14* are the longest, among the members, possessing five exons and four introns (Fig. 4b). A characteristic feature of this homeolog (present on A4 and B4 chromosomes, respectively) is the presence of a long intron between exon 2 and exon 3. Many members of this family have homeologous pair with similar characteristics. For example, *AhRS1* and *AhRS12*, with three exons and two introns are present on chromosome A1 and B1, respectively; *AhRS2* and *AhRS13* on chromosome A3 and B3 (Fig. 4a), having 13 exons and 12 introns; *AhRS9* and *AhRS17*, on chromosome A7 and B7, having 12 exons and 11 introns; *AhRS6* and *AhRS16* on chromosome A6 and B6, having 13 exons and 12 introns in approximately 4 kb region. *AhRS7* and *AhRS8* showed high sequence similarity and are present on chromosome A7 but have structural difference having an extra exon on *AhRS8*. *AhRS10* and *AhRS11*, present in chromosome A7 and A9, respectively, contain a single exon only.

Similarly, seven possible *SS* gene members have been predicted in *A. hypogaea* and the members are distributed majorly on chromosomes A6 and B6 but some variants have also been found in chromosomes A1, A7, B3, and B5 (Fig. 4a). This gene catalyzes the conversion of Raffinose to Stachyose by addition of a galactose residue from galactinol. The members can also play diverse roles, undergoing galactinol independent reactions and ciceritol biosynthesis. As revealed by the phylogenetic tree (Fig. 4b), *AhSS5* (chromosome B3) and *AhSS6* (chromosome B5) are highly similar but *AhSS6* and *AhSS5* have one and three exons respectively. *AhSS5* was not found in the studied transcriptomic data and the expression of *AhSS6* was also found very low. *AhSS1* and *AhSS4* have single exon and is located on chromosome A1 and A7. *AhSS4* was not found in the seed and *AhSS1* expression was also found significantly lower. *AhSS3*/*AhSS8* pair and *AhSS2*/*AhSS7* pair on chromosome A6/B6 (Fig. 4a) are examples of homeologous genes, with four exons and three introns, showing high sequence similarity. Three conserved motifs were also found in all members of RFOs family (Fig. 4c) with the statistically overrepresented sequences, as shown in Fig. 4d.

### Expression of the identified genes in different seed maturity stages and leaves of contrasting varieties

**Figure 5:**
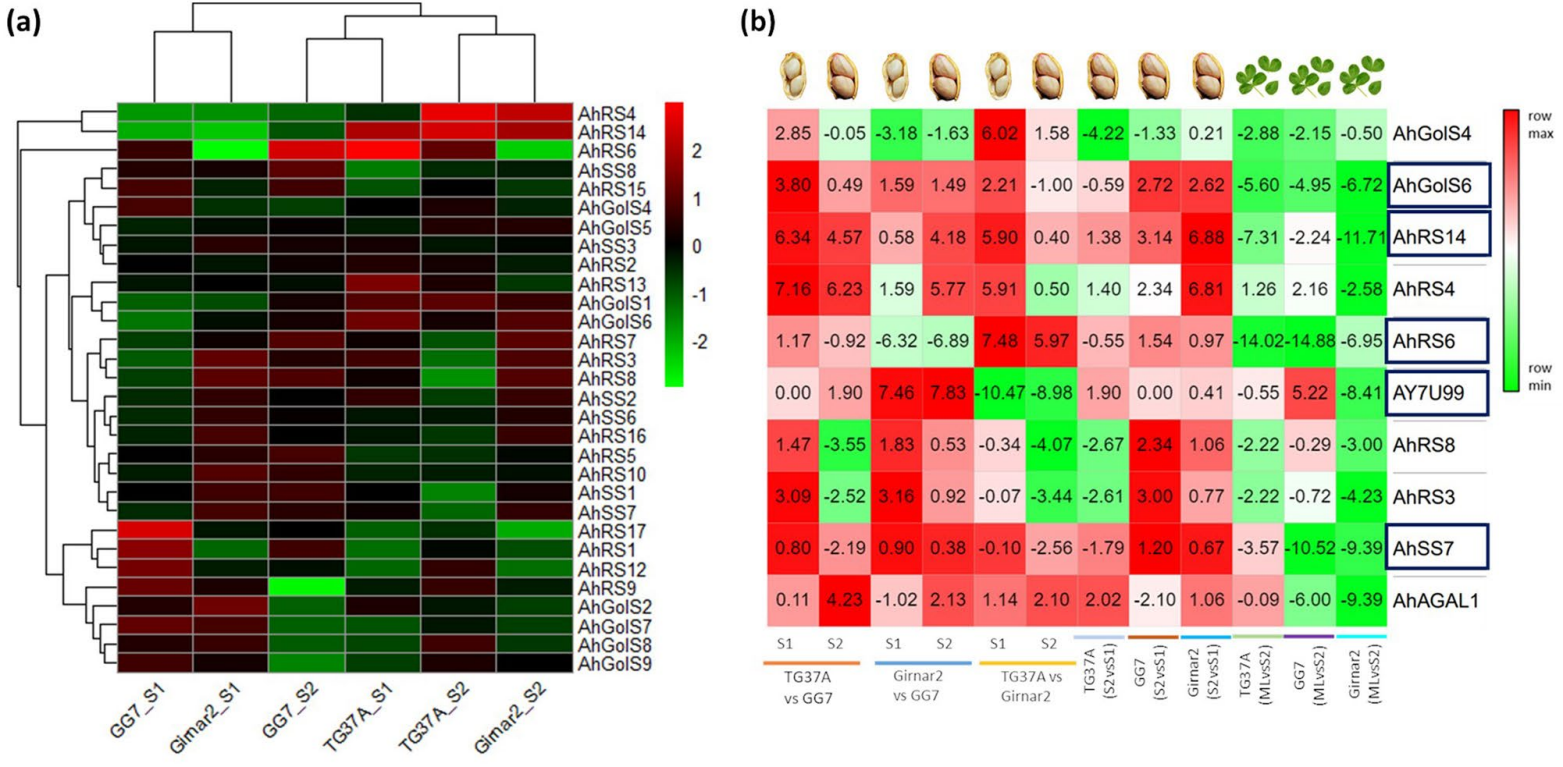
Expression profile of differentially expressed genes related to RFOs biosynthesis across the varieties and seed stages. **(a)** Global heatmap of RFOs genes. Upregulation and downregulation is indicated by the intensity of red and green respectively. FPKM values have been used for designing the heatmap. The figure has been created using ‘gplots’ package 3.1.1 version of the R software. **(b)** Heatmap of log2 fold change values across various combinations. The name of genes is represented on Y-axis and X-axis represents varieties and stages. The figure has been created using Morpheus web tool (https://software.broadinstitute.org/morpheus).

Expression statistics from RNA-seq data revealed significant expression of two *GolS* genes (*AhGolS4* and *AhGolS6*) among the contrasting varieties (Fig. 5a). *AhGolS4* showed downregulation at S2 in low RFOs variety (GG7) and upregulation in high RFOs variety (TG37A and Girnar 2). *AhGolS6* showed the opposite effect with overexpression in GG7 and downregulation in TG37A during the matured seed (S2) stage. Genes like *AhGolS1* and *AhGolS5*, situated on chromosome A9/B9 were not considered differentially expressed despite their high transcript abundance. *AhGolS2*, *AhGolS3*, *AhGolS7*, and *AhGolS9* were also identified in the transcriptome data but with low expression profiles. However, expression of *AhGolS5* and *AhGolS8* were not recorded in the studied RNA-seq data. Maximum members from raffinose synthase family showed significant differential expression. *AhRS1* and *AhRS12* had very low expression in the high RFOs varieties (TG37A and Girnar 2) but a reduction was found at S2 of the low RFOs variety (GG7). *AhRS4* and *AhRS14*, on the other hand, were expressed in high RFOs varieties, with significant increase at S2, while very low transcript density was recorded in low RFOs variety. *AhRS6* and *AhRS16* expression was almost constant among the seed stages in Girnar 2 and a non-significant upregulation and downregulation were seen in GG7 and TG37A, respectively. A high transcript density was obtained for both the genes across the varieties, however, the transcript density of *AhRS6* was significantly lower (333) as compared to *AhRS16* (46,672) in Girnar 2. *AhRS9* and *AhRS17* showed significant downregulation at S2 of GG7 while a non-significant upregulation was seen in TG37A for *AhRS9* (Fig. 5b). *AhRS2* and *AhRS13* showed a very low level of expression in GG7 and Girnar 2, while, it was non-significantly downregulated at TG37A S2. *AhRS10* and *AhRS11* had almost nil expression in the seed, while *AhRS8* and *AhRS3* showed constant expression in Girnar 2 but upregulation in GG7 and downregulation in TG37A. *AhRS5* and *AhRS15* were generally expressed in stem and their expression in the seeds was low and showed constant expression across stages. Among the *SS* family genes, *AhSS2*/*AhSS7* possibly play the regulatory role in seed RFOs biosynthesis, with a trend showing upregulation at matured seed (S2) stage in GG7 and Girnar 2 and downregulation in TG37A. Expression of *AhSS6* was very low with slightly higher transcript density at S2 in GG7 and Girnar 2 (Fig. 5a). In TG37A, S2 exhibited a reduced transcript density, while *AhSS3*/*AhSS8* pair showed very low expression in the studied seed samples.

### Validation of RNA-sequencing results in contrasting peanut varieties

**Figure 6:**
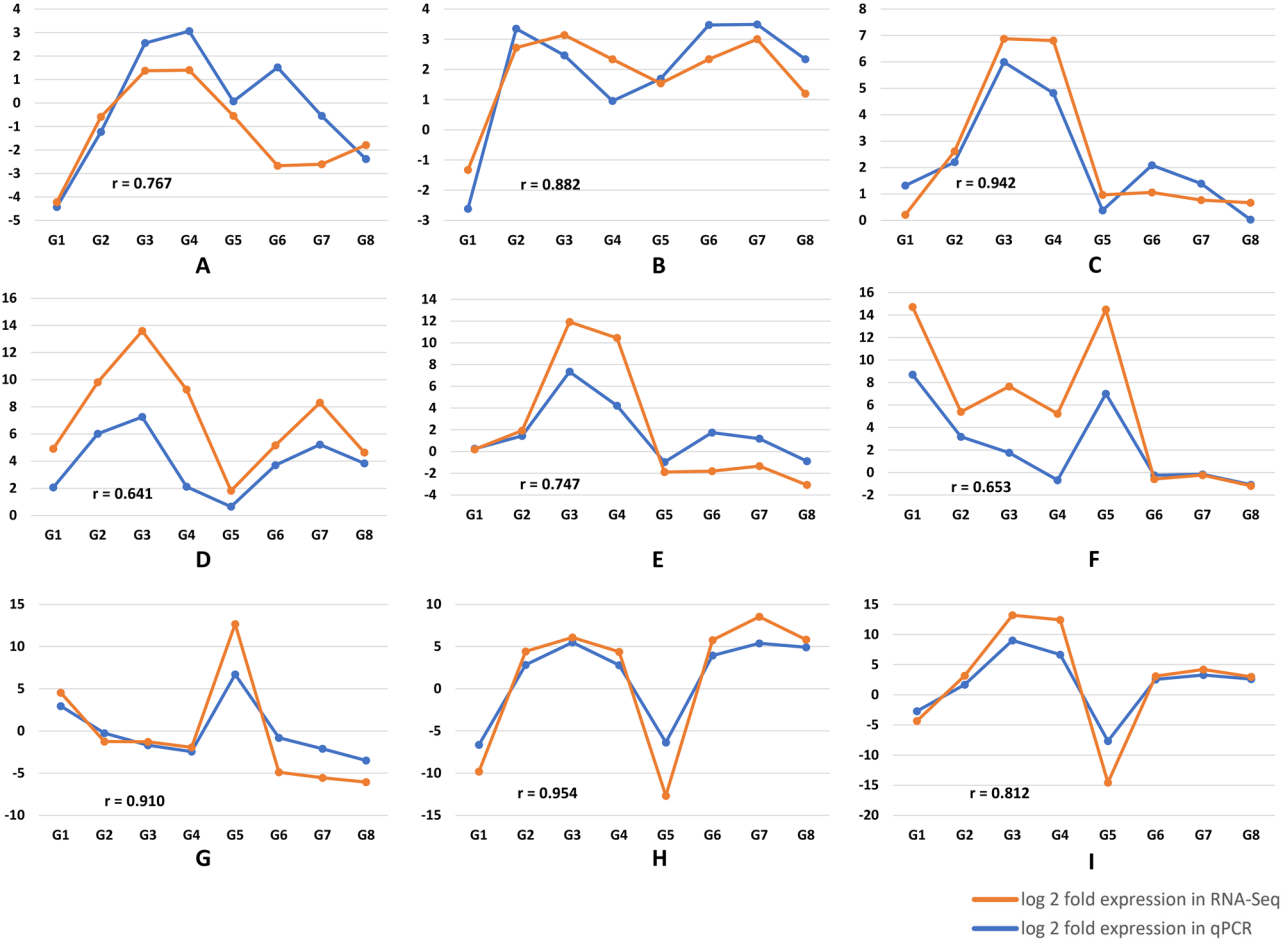
Graphs showing the correlation of log 2 FC of RFOs related genes in qPCR to that of RNA-seq data**.** A-I represents different sample combinations while G1-G8 represents different genes. A = TG37A (S2 vs S1), B = GG7 (S2 vs S1), C = Girnar 2 (S2 vs S1), D = TG37A vs GG7 (S1), E = TG37A vs GG7 (S2), F = TG37A vs Girnar 2 (S1), G = TG37A vs Girnar 2 (S2). H = Girnar 2 vs GG7 (S1), I = Girnar 2 vs GG7 (S2). G1 = AhGolS4, G2 = AhGolS6, G3 = AhRS14, G4 = AhRS4, G5 = AhRS6, G6 = AhRS8, G7 = AhRS3, G8 = AhSS7.

For validation of RNA-seq data at two maturity stages (S1 and S2), ten genes were identified having significant role for RFOs accumulation. With *Ubiquitin 1* (*UBI 1*) as the endogenous control, the genes were validated across the maturing (S1) and matured (S2) seed stage of the Spanish (TG37A and GG7) and Virginia (Girnar 2) varieties. To confirm the seed specific expression, the genes were also validated in the matured leaf stage of these contrasting varieties. The galactinol synthase gene (*AhGolS4* and *AhGolS6*) showed significant upregulation in maturing seed (S1) stage of TG37A, when compared to GG7 or Girnar 2. *AhGolS4* showed significant downregulation at S1 of Girnar 2, S2 of TG37A and in the leaves of the Spanish varieties (Fig. 5b). *AhGolS6* was also upregulated in S2 as compared to S1 for GG7 and Girnar 2. Interestingly, a significant downregulation in leaf samples of all the varieties was also observed. *AhRS14*, a raffinose synthase family gene, exhibited upregulation in all the seed samples including TG37A vs GG7 S1 (log2FC = 6.34) and S2 (log2FC = 4.58), Girnar 2 vs GG7 S2 (log2FC = 4.18), TG37A vs Girnar 2 S1 (log2FC = 5.90), S2 of GG7 (log2FC = 3.14) and Girnar 2 S1 (log2FC = 6.88). *AhRS14* showed downregulation in all the leaf samples (Fig. 5b), while *AhRS4* showed upregulation, with very high fold expression in TG37A vs GG7 S1 (log2FC = 7.17) and S2 (log2FC = 6.23), Girnar 2 vs GG7 S2 (log2FC = 5.77), TG37A vs Girnar 2 S1 (log2FC = 5.91), and Girnar 2 S2 (log2FC = 6.81). *AhRS4* showed upregulation in the leaf of Spanish varieties, while Virginia peanut varieties showed downregulation. *AhRS6* was highly downregulated in leaf samples and exhibited very high upregulation among the Spanish varieties, when compared to the Virginia variety. *AhRSV* or *AY7U99* gene is probably specific to Virginia variety (Girnar 2), showing significant upregulation in S1 and S2 when compared to its Spanish counterparts. However, significant downregulation of this gene in the leaves of the Girnar 2 indicated its seed specificity. *AhRS8* was found upregulated in S2 of GG7 when compared to S1, while downregulation was recorded in matured seeds of TG37A vs GG7, TG37A vs Girnar 2, and TG37A. The leaf samples of TG37A and Girnar 2 also showed significant downregulation for this gene (Fig. 5b). *AhRS3* exhibited overexpression at S1, while downregulation at S2 stage of TG37A vs GG7. Overexpression was also observed at S1 of Girnar 2 vs GG7 and S2 of GG7 as compared to S1. *AhRS3* was found downregulated at S2 stage of TG37A vs Girnar 2 and TG37A itself and was also found downregulated in the leaf samples in TG37A and Girnar 2. *AhSS7*, a gene from stachyose synthase family, showed downregulation at S2 of TG37A vs GG7, TG37A vs Girnar 2 and the leaf samples of all varieties as compared to their seeds. There seems to be no significant upregulation in any of the combinations for this gene (Fig. 5b). The comparison of fold change obtained via real-time PCR and that of RNA-seq study revealed a positive correlation across samples (Fig. 6). Genes like *AhGolS1* and *AhGolS5*, on chromosome A9/B9 were not considered differentially expressed (despite their high transcript abundance) as they were constitutively expressed across the seed stages, with a tendency to increase towards maturity. *AhGolS2*, *AhGolS3*, *AhGolS7*, and *AhGolS9* were also identified in the transcriptome data but they had very low expression profiles across varieties. This can be due to the expression of these genes at sites other than seed, viz., root tip, leaf, pericarp, and early embryo, respectively. *AhGolS5* was not found despite its expression in seed and *AhGolS8* was absent, probably due to its leaf-specific expression, as reported in the peanutbase database. (https://peanutbase.org/feature/Arachis/hypogaea/gene/arahy.Tifrunner.gnm1.ann1.96F7ZV). *AhSS4* was not found in seed and *AhSS1* expression was also significantly lower. The expression atlas of *AhSS1*, *AhSS4*, *AhSS5*, and *AhSS6* showing expression at florescence, leaf, leaf + salicylic acid, and all tissues, respectively, can be the reason for low expression in seed tissues. *AhSS3*/*AhSS8* pair had almost nil expression in seed samples, which is also evident from the leaf-specific expression in the expression atlas.

## Discussion

Peanut, despite being a nutrient-dense crop with hundreds of bioactive compounds and a wide range of usage in industry (cattle and poultry feed, oilcake) and household (as pulse as well as oilseed), faces lower consumer acceptance due to the problem of flatulence. For wider consumer acceptability and reduced negative impact as cattle feed, RFOs must be reduced. As seeds are the economic part of peanuts, identification of candidate genes in the seeds can pave the way towards low RFOs crop development programs. This study revealed the diversity in RFOs content and composition among the varieties and also within the plant developmental stages. As the change can be associated with the environmental conditions^33^, we measured the total RFOs, ranging from 0.4–0.67%, which is in accordance with previous reports^34,35^. During the seed developmental stages, RFOs accumulation showed an increasing trend towards maturity, indicating its role as osmoprotectants in seed desiccation tolerance^36^. Ion-exchange chromatography showed stachyose to have the maximum contribution to seed RFOs, which is in accordance with previous reports^35^. Higher sucrose concentration also increases the accumulation of raffinose^37^, which is evident from the positive correlation (r = 0.64) found in this study.

**Figure 7:**
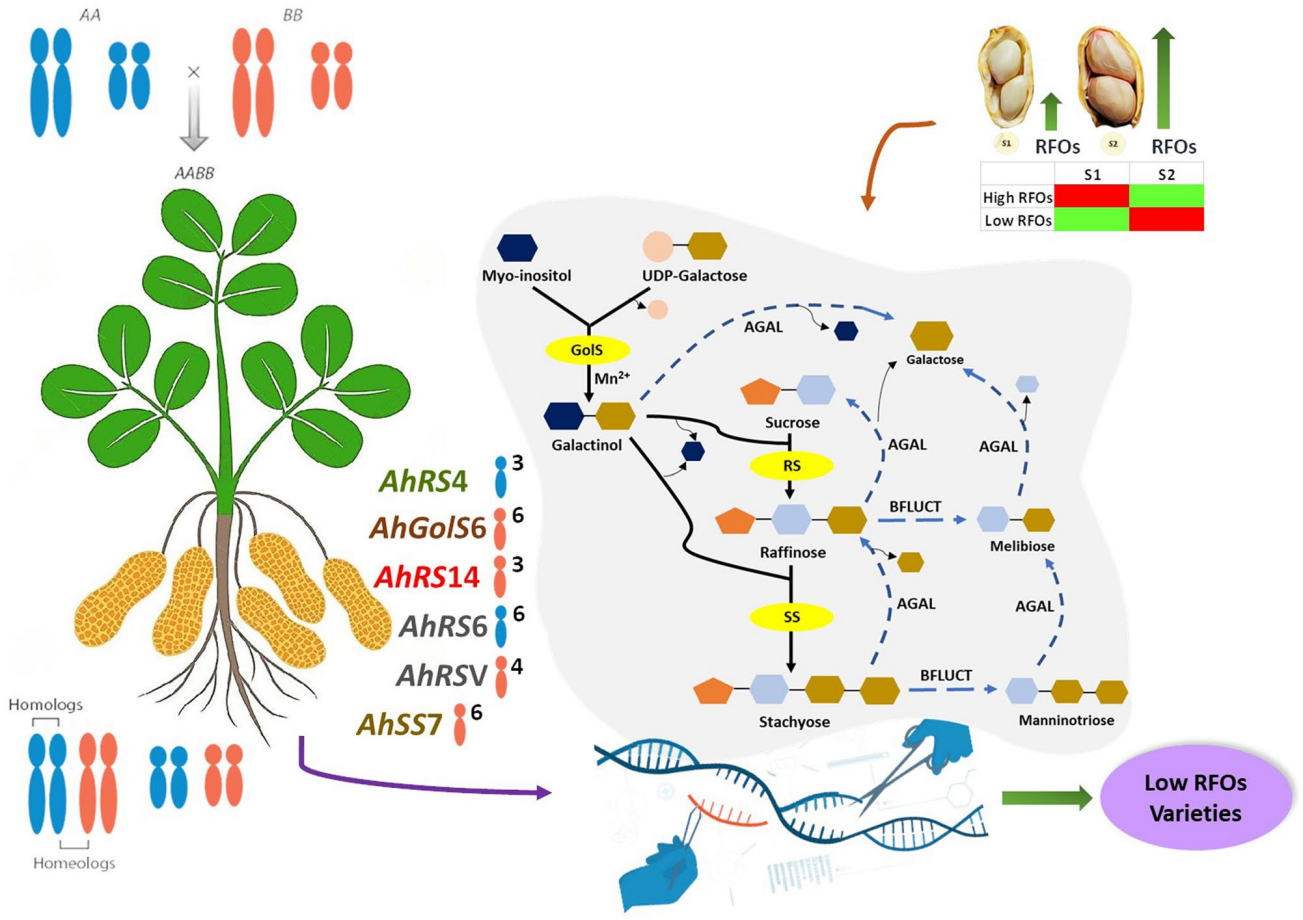
A model depicting the spatio-temporal and selective expression pattern of the genes involved in RFOs accumulation in peanuts. The genes shows upregulation (red) at early stage of seed development (S1) in high RFOs varieties and at later stage (S1) in low RFOs varieties. The RFOs pathway is represented with key genes, namely, galactinol synthase (*GolS*), raffinose synthase (*RS*) and stachyose synthase (*SS*) , which are highlighted (). Alpha galactosidase (AGAL) and beta fructofuranosidase (BFLUCT) are the RFOs degrading enzymes. All the genes are highly expressed in the seeds but *AhRS4* also shows expression in leaves. The location of these genes is represented by the chromosome number as superscript to the genome, A () or B (). These genes can be targeted to develop low RFOs peanut varieties.

Previous reports suggested *GolS* as the regulatory gene in RFOs biosynthesis^28,38^, on the contrary, this study found significant expression of *RS* genes within and across contrast varieties (Fig. 7). *GolS* and *SS* rather showed constitutive expression in seeds as well as in leaves. Some studies reported the accumulation of *RS* transcripts at a later stage of seed maturation^39,40^, which is consistent with this study. Although *RS* plays a regulatory role, the amount of stachyose (not raffinose) increased in the matured seed which may be accounted for the transient expression of *RS* and the subsequent product inhibition^41^ in dicots, resulting in rapid conversion of raffinose to stachyose. A unique transcript of *RS* gene (*AhRSV* or *AY7U99*) was identified on PeanutBase database, which was absent in the Peanut Genome Resources. This could be due to the kind of genome assembly used for the database development. PeanutBase uses a high-quality genome assembly for peanut cultivar "Tifrunner", which is a Virginia group cultivar^42^ while Peanut Genome Resource uses A. *hypogaea* var. *Shitouqi*, a cultivar belonging to the Spanish group^43^. The RNA-seq data could not find any transcript of this gene in Spanish variety (TG37A and GG7) and the qPCR data also showed non-significant expression between seed stages (S2vsS1) in TG37A and GG7 varieties. However, on comparing TG37A vs GG7, differential expression at S1 and S2 stages was observed. This can be due to the little amount of expression in seeds of TG37A (high RFOs) with almost nil expression in GG7 (low RFOs). However, this does not preclude the specificity of this gene for the Virginia group, as is evident from the expression values. The gene is constantly expressed in Girnar 2, particularly in the seeds. *AhRSV* showed very high upregulation in Girnar 2 when compared to the Spanish varieties at both the seed stages. The absence of a band in agarose gel (Supplementary Fig. S4a online) after semi-quantitative PCR for the seed samples of Spanish group varieties and leaf samples of all the varieties also suggests the possibility of this gene as a seed specific biomarker for Virginia varieties. No orthologs of galactan-galactan galactosyl transferase (*GGT*) enzyme was found in the in-silico analysis. Furthermore, RNA-seq data also ruled out the possibility of galactinol independent RFOs biosynthesis in peanut seeds. Genes related to RFOs biosynthesis were found upregulated during early stage of seed development in high RFOs variety (TG37A), providing a wider window for RFOs accumulation. In the later stage, these genes get downregulated with a subsequent increase in genes encoding phospholipid:diacylglycerol acyltransferase (PDAT) and mono-galactosyl diacyl glycerol (MGDG), indicating the role of RFOs in providing carbon skeleton to oil synthesis or thylakoid membrane biogenesis. Reports on oil accumulation during fast accumulation stage in peanut, which occurs towards the later part of seed development also supports this observation^44^. For low RFOs variety (GG7), the same genes get upregulated at S2, allowing a smaller window for accumulation. RFOs may get accumulated to a certain threshold and activate RFOs biosynthetic reactions in TG37A. Reports also suggest maturing seeds to accumulate compounds that are remobilized for post-germinative seedling establishment^45^. When the shortage of RFOs is detected by receptors in low RFOs variety (GG7), biosynthetic genes get upregulated transiently, synthesizing RFOs, at the later stage (S2) of seed maturation. Thus, even when RFOs biosynthetic genes get downregulated at S2 of TG37A, the upregulated genes at S2 of GG7 hardly meet the threshold.

Genotypic and stage-specific differential gene expression in peanut during seed maturity was also found in this study. Upregulation of galactinol synthase gene (*AhGolS4*) in maturing seed stage (S1) of high RFOs Spanish variety (TG37A) and significant downregulation in its Virginia counterpart (Girnar 2), makes it a major player in RFOs accumulation in varieties belonging to Spanish group. Seed specific expression of *AhGolS6* at S1 of TG37A and similar upregulation at S2 of GG7 and Girnar 2 suggests its role throughout the seed development (Fig. 5b). Among the *RS* genes, *AhRS14* showed significant upregulation at both seed stages of TG37A when compared to GG7, even after log2 fold change of 3 in GG7_S2. Expression of *AhRS14* remained significantly high in TG37A_S1 but drastic increase in expression in Girnar 2_S2, makes it comparable to TG37A. High RFOs groups (TG37A and Girnar 2) exhibit much higher *AhRS14* expression at S2 as compared to low RFOs variety (GG7). The activity of *AhRS14*, specifically in seeds of high RFOs groups is also confirmed by very high downregulation in the leaves (Fig. 5b) and absence of band in the agarose gel electrophoresis (Supplementary Fig. S4b online)**.** *AhRS4* also showed constant high expression in TG37A in both seed stages and at S2 of Girnar 2, making it an important DEG for RFOs accumulation. However, the expression is not specific to seed and it seems to play an important role throughout the plant (Supplementary Fig. S4b online). *AhRS6* is not a differentiating factor in high and low RFOs variety but acts as a candidate when two groups (Spanish and Virginia) are compared. Spanish varieties showed nearly seven fold upregulation in both the seed stages when compared to the Virginia variety. It is also highly specific to RFOs biosynthesis in seeds because, in leaves, it shows very high downregulation. As far as *SS* genes are concerned, only one member (*AhSS7*) had significant downregulation in leaves and showed an almost constitutive expression across the seed stages. This suggests the regulatory role of *RS* genes in accumulating RFOs in seed with *GolS* and *SS*, showing a constant expression.

This study identified many homeologs of RFOs biosynthetic family, most of which are situated on chromosome 6 (A6/B6), namely, *AhGolS1* and *AhGolS5*, *AhRS6 and AhRS16*, *AhSS2* and *AhSS7*, *AhSS3* and *AhSS8*. Maximum number of homeologs have been identified in *RS* family at chromosome A1/B1 (*AhRS1* and *AhRS12*), A3/B3 (*AhRS4* and *AhRS14*, *AhRS2* and *AhRS13*), A5/B5 (*AhRS5* and *AhRS15*) and A7/B7 (*AhRS9* and *AhRS17*). Homeologs on chromosome A6/B6 showed constitutive expression and high transcript density, for which most of them were not considered DEGs. Although, *AhRS6* and *AhRS16* have high FPKM, only *AhRS6* (from A genome) was considered to have significant differential expression while *AhRS16* (from B genome) was not. *AhRS4* and *AhRS14* showed significant upregulation at S2 but the trend was reversed in *AhRS2*/*AhRS13* pair while being present on the same chromosome (A3/B3). Among *AhRS5*/*AhRS15*, located on chromosome A5/B5, *AhRS15* (from B genome) showed significant expression. Similarly, *AhRS17* (from B7) also showed significant expression over *AhRS9* (from A7). Also, among *AhSS2* and *AhSS7*, only *AhSS7* (from B genome) showed significant differential expression. This selective expression pattern of the homeologs strongly hints towards the evolutionary role of these genes during allopolyploidization of peanuts, as represented in the graphical summary (Fig. 7).

## Conclusion

In this study, nine genes were found associated with RFOs biosynthesis in peanut seeds and of these, five genes namely, *AhGolS6*, *AhRS14*, *AhRS6*, *AhRS*V and *AhSS7* were found specific to the seeds. *AhRS4* also played a potential role in differentiating high and low RFOs varieties but it was not specific to seed. *AhSS7* is important for overall RFOs biosynthesis due to its constitutive expression in seed however, it does not play a regulatory role in RFOs biosynthesis. *AhRS14* seems to play the regulatory role in contrast varieties while *AhRS6* is vital for studying RFOs variation among the groups. The possibility of *AhRSV* as the biomarker for group identification should further be validated in a larger population of Spanish and Virginia varieties. The higher content of RFOs in Virginia varieties, in general, can also be attributed to *AhRSV*. The evolutionary aspect of selective expression of RFOs biosynthetic genes can be further studied to explore the functions of such genes before and after allopolyploidization. The seed-specific candidate genes can further be functionally validated and targeted to generate low RFOs varieties with better consumer preference. In the future, through advanced molecular tools and techniques, specific candidate genes can be identified which could be targeted to develop peanut varieties with enhanced nutritional qualities and climate resilience.

## Materials and methods

It has been confirmed that the experimental samples of plants, including the collection of plant material, complied with relevant institutional, national, and international guidelines and legislation with appropriate permissions from Institute authorities of ICAR-Indian Agricultural Research Institute, Ranchi, Jharkhand, India for collection of plant specimens.

### Plant material

Six peanut varieties (Girnar 3, Girnar 2, GG-5, GG-7, GG-20, TG-37A) representing a diversity of seed RFOs content were used in this study^35,46^. The seeds were obtained from ICAR-Directorate of Groundnut Research, Junagadh, India and were grown at ICAR-IIAB farm, Ranchi, Jharkhand, India (Latitude: 23°16′26.8"N, Longitude: 85°20′30.9"E, Altitude: 655 m AMSL) during 2020–21. Seeds were first treated with Bavistin (0.5%) and then sown in 60 pots (ten pots per variety) having growth-media consisting of coco peat: vermiculite: sand (1:2:1), at a depth of 5.0 cm, in a polyhouse. Light but frequent irrigation during pegging and pod development was practiced to promote the profuse flowering and seed set.

### RFOs profile analysis

Peanut seed soluble carbohydrates were extracted with 80% (v/v) ethanol using standard methods^47^. Inositol, glucose, fructose, raffinose and stachyose content were measured using an ion chromatograph (Dionex, ICS 3000) equipped with amino trap column, CarboPac PA10 guard column and analytical column following method outlined by^35^ and chromatograms were generated (Supplementary Fig. S1 online).

### Selection of contrasting varieties and sample collection

Based on the chromatographic data, three varieties were selected, namely, TG37A (High RFOs, Spanish type), GG7 (Low RFOs, Spanish type), and Girnar 2 (High RFOs, Virginia type). Pod and seed development stages were determined by tagging individual flowers at anthesis using two different colored tags on a single plant, signifying the variation in flowering, anthesis, and pod development. Tagging was performed 40 days after sowing (DAS) in Girnar 2 while for GG7 and TG37A, it was 35 and 30 DAS, respectively. Collection of the maturing stage (S1) seed was done 55 days after anthesis (DAA) from all the varieties, followed by matured stage (S2) seed collection at 70 DAA. S1 seeds were identified by their soft texture, whitish seeds and absence of cracking/rattling sound while S2 seeds had well defined ridges on pod, pink seeds and made a rattling sound whenever shaken. At each harvest, pods with a similar tag colour were selected for further downstream analysis. A set of seeds were dipped in RNAlater® solution and immediately stored at -80 °C before RNA extraction. While another set was taken in ice-packs for enzyme-based RFOs quantification. A total of 18 samples were used for RNA-seq analysis which consisted of TG37A maturing seed (TG37A_S1), TG37A matured seed (TG37A_S2), GG7 maturing seed (GG7_S1), GG7 matured seed (GG7_S2), Girnar 2 maturing seed (Girnar2_S1), and Girnar 2 matured seed (Girnar2_S2). The samples were collected in three biological replications each.

### Enzyme-based quantification of RFOs in seeds

Seed meal of weighed quantity was transferred to a thimble and extracted in 150 ml hexane for 12 h using the Soxhlet extraction assembly^48^. Quantification and extraction of oligosaccharides (glucose, sucrose, RFOs) from the defatted meal was performed using Megazyme kit (Megazyme International Ireland Ltd., Bray, Ireland cat. no. K-RAFGL 05/2008).

### RNA extraction, library construction and illumina sequencing

Total RNA was extracted from frozen seed samples dipped in RNAlater® using RNeasy Plant Mini Kit (Qiagen, Hilden, Germany) (Supplementary Fig. S2 online). mRNA was enriched using the NEBNext Poly (A) mRNA magnetic isolation module (Catalog: E7490, New England Biolabs). The NEBNext® UltraTM II RNA Library Prep Kit for Illumina (Catalog: E7775S, New England Biolabs) was used to prepare the libraries from the enriched mRNAs. The resulting double-stranded cDNA fragments were purified using 1.8X AMPure XP beads (Catalog: A63881, Beckman Coulter) and PCR amplified (10 cycles). Library concentration was evaluated in Qubit™ 3 Fluorometer (Catalog: Q33216, Life Technologies) (Supplementary Table S1 online) and it’s quality was assessed using Agilent 4150 TapeStation system (Catalog: G2992AA, Agilent) (Supplementary Fig. S3 online). RNA sequencing of the libraries was performed using Illumina Novaseq 6000 at Clevergene Biocorp Private Limited (Bengaluru, Karnataka, India). The quality of the raw data obtained by paired-end sequencing was analyzed using FastQC (https://www.bioinformatics.babraham.ac.uk/projects/fastqc/) and MultiQC^49^ software. The low-quality reads and the adaptor contaminations were removed using Trimmomatic v0.32 software^50^.

### Alignment and expression analysis

The QC passed reads were mapped onto the indexed peanut reference genome (*Arachis hypogaea cv. Tifrunner*, https://peanutbase.org/data/v2/Arachis/hypogaea/genomes/Tifrunner.gnm1.KYV3/] using TopHat^51^ aligner. Transcript compilation, gene identification, and gene level expression values (FPKM ) were calculated using Cufflinks package^52,53^. Expression similarity between biological replicates was checked by spearman correlation and principal components analysis (PCA). Differential expression (DE) analysis was performed using edgeR (http://www.bioconductor.org/packages/2.12/bioc/html/edgeR.html)^54^. Heatmaps were generated using gplots package 3.1.1 version of ‘R’ program^55^ and Morpheus web tool (https://software.broadinstitute.org/morpheus).

### Gene ontology (GO) analysis

Transcript annotation was done using Blast2GO functional annotation workflow available in OmicsBox version 1.3.11 (https://www.biobam.com/omicsbox/) and sorted transcripts were blasted against the non-redundant NCBI protein sequences (nr v5) database using the blastx-fast program with e-value 1.0E-10. The BLAST results were subjected to InterProScan using the EMBL-EBI database and GO mapping^56^ and annotation were carried out using the COG database. All the retrieved GO terms were combined with the GO annotation to find the reliable plant generic GO terms. The Kyoto Encyclopedia of Genes and Genomics (KEGG)^57^ pathways were established with the OmicsBox 1.3.11 software using the KEGG pathway tool with default settings.

### Expression profile analysis of genes and validation of RNA-Seq data

Conventional PCR was performed in a total volume of 25 μL using Taq DNA Polymerase and Taq PCR Core Kit (Qiagen, Hilden, Germany) in a Mastercycler® Nexus thermal cycler (Eppendorf, Germany). qPCR was performed using SYBR select Master Mix (Thermo Fisher Scientific, Massachusetts, USA) in a StepOnePlus™ Real-Time PCR System (Applied Biosystems, USA) with gene-specific primers. These primers were designed using PrimerQuest tool (PrimerQuest® program, IDT, Coralville, Iowa, USA, https://www.idtdna.com/SciTools) with custom parameters in Primer Force Location Settings (Supplementary Table S2 online). Analysis of candidate genes was performed using RefFinder software program (https://www.heartcure.com.au/reffinder/) (Supplementary Table S3 online). Livak’s 2^−ΔΔCT^ method^58^ was used for the analysis.

### In silico characterization of candidate genes

Sequence information of the genes related to RFOs metabolism were obtained from PeanutBase (https://peanutbase.org/home)^42^ database using keywords like “galactinol synthase”, “stachyose synthase”, “galactinol-sucrose galactosyltransferase” “Alpha galactosidase”, and “beta fructofuranosidase”. Gene names were allotted based on their location in *Arachis* chromosome (ascending order) and chromosome sizes were extracted from previous literature^43^ (Supplementary Table S4 online). Mapping the genes on the peanut chromosome was done using MapChart^59^ and *A. hypogaea* cv. Shitouqi (zh.h0235)^43^ was used for chromosome localization study (Supplementary Table S4 online). Phylogenetic tree and gene structure visualization was done using Gene Structure Display Server (GSDS)^60^ while motif composition of each gene was identified using Multiple EM For Motif Elicitation (MEME) tool^61^.

### Statistical analysis

Analysis of variance (ANOVA) and Duncan's Multiple Range Test (DMRT) was performed using DSAASTAT 1.1^62^, while correlation between different traits was studied using Microsoft Excel Analysis ToolPak program. The results having *p*-value ≤ 0.05 were considered significant. Livak’s 2^−ΔΔCT^ method^58^ was used for the analysing the relative changes in gene expression from qPCR experiment.

## Supplementary Information

Supplementary Information.

## Data Availability

RNA-seq data generated in the study have been deposited in the National Centre for Biotechnology Information (NCBI) under BioProject ID PRJNA895972, which can be accessed via https://www.ncbi.nlm.nih.gov/bioproject/PRJNA895972
