# Supplementary material for: Spatio-temporal expression pattern of Raffinose Synthase genes determine the levels of Raffinose Family Oligosaccharides in peanut (Arachis hypogaea L.) seed
Source: Sci Rep. 2023 Jan 16;13:795. doi: 10.1038/s41598-023-27890-z (PMC9842710; doi:10.1038/s41598-023-27890-z)
Supplement: Supplementary file 1 — Supplementary Information. [file 41598_2023_27890_MOESM1_ESM.pdf]

Supplementary Information

Spatio-temporal expression pattern of Raffinose Synthase genes determine the levels of Raffinose Family Oligosaccharides in peanut (*Arachis hypogaea* L.) seed

Rajarshi Sanyal [1,2], Bhubaneswar Pradhan[1,#], Danish Md. Jawed[1], Kishor U. Tribhuvan[1], Anil Dahuja[2], Madan Kumar[1], Narendra Kumar[3], Gyan P. Mishra[4], Chet Ram[5], Mahesh Kumar Mahatma[6], Binay K. Singh[1], Satendra K. Mangrauthia[7], Anil K. Singh[8], T.R. Sharma[1, ##], Arunava Pattanayak[1], and Sujit K. Bishi[1]*

[1] School of Genomics and Molecular Breeding, ICAR–Indian Institute of Agricultural Biotechnology, Garhkhatanga, Ranchi-834003, India

[2] Division of Biochemistry, ICAR-Indian Agricultural Research Institute. New Delhi-110012, India

[3] Regional Research Station, ICAR-Directorate of Groundnut Research, Bikaner-334006, India

[4] Division of Genetics, ICAR-Indian Agricultural Research Institute. New Delhi-110012, India

[5] ICAR-Central Institute for Arid Horticulture, Beechwal, Bikaner-334006, India

[6] ICAR-National Research Centre on Seed Spices, Beawar Road, Ajmer-305206, India

[7] ICAR-Indian Institute of Rice Research, Hyderabad-500030, India

[8] ICAR-National Institute of Plant Biotechnology, New Delhi-110012, India

Present address

[#] Department of Agricultural Biotechnology, Faculty Centre for Integrated Rural Development and Management, Ramakrishna Mission Vivekananda Educational and Research Institute, Kolkata-700103, India

[##] Career Point University, Alaniya, Kota-325003, India

Corresponding author: Sujit K Bishi

Table S1. Complete library QC status of the peanut samples

Sl. No.	Well	Sample ID	Qubit conc. (ng/µl)	Size (bp)	Conc. (nmol)
1	B2	TG37A_S1_R1	13.1	530	37.45
2	C1	TG37A_S1_R2	11.8	498	35.90
3	A2	TG37A_S1_R3	8	541	22.41
4	B1	TG37A_S2_R1	8.72	493	26.80
5	C3	TG37A_S2_R2	3.82	505	11.46
6	H1	TG37A_S2_R3	7.64	536	21.60
7	G2	Girnar2_S1_R1	4.36	548	12.05
8	B3	Girnar2_S1_R2	29.2	503	87.96
9	E1	Girnar2_S1_R3	11	536	31.09
10	H2	Girnar2_S2_R1	7.26	509	21.61
11	A3	Girnar2_S2_R2	18.9	540	53.03
12	D1	Girnar2_S2_R3	9.5	509	28.28
13	F2	GG7_S1_R1	43.4	512	128.43
14	D2	GG7_S1_R2	23.4	547	64.82
15	C2	GG7_S1_R3	7.38	522	21.42
16	E2	GG7_S2_R1	19.9	503	59.94
17	G1	GG7_S2_R2	20.2	547	55.95
18	F1	GG7_S2_R3	18.2	527	52.33

Qubit concentrations above 2ng/µl or 10nmol signifies the permissible range. Data was analyzed using TapeStation Analysis Software 3.2 in Agilent 4150 TapeStation System. R1, R2 and R3 indicates the three replications in each sample.

Table S2. Details of the primers selected for reference gene identification and qPCR analysis

Sl. No.	Gene name	Tentative annotation	Primer sequence	Amplicon length (bp)
1	*ADH3*	Alcohol dehydrogenase class III	F: GACGCTTGGCGAGATCAACA<br>R: AACCGGACAACCACCACATG	140
2	*ACTIN11*	Actin 11	F: ATGCTAGTGGTCGTACAACTGG<br>R: CTAGACGAAGGATAGCATGTGG	108
3	*ELF1B*	Elongation factor1-beta	F: AAGCTTCCCTGGCAAAGCTCAA<br>R: TTCCTCAGCTGCCTTCTTATCC	154
4	*G6PD*	Glucose 6-phosphate-dehydrogenase	F: ACCATTCCAGAGGCTTATGAGC<br>R: AAGGGAGTGACTTGAACTCTCC	151
5	*ACTIN1*	Actin1	F: TGGTCTCGGTTTCCTGAGTT<br>R: AATACCACTCCAAAGCAAACG	114
6	*UBI1*	Polyubiquitin	F: TCTTGTCCTCCGTCTTAGGG<br>R: AGCAAGGGTCCTTCCATCTT	196
7	*UBC1*	Ubiquitin C	F: AAGCCGAAGAAGATCAAGCAC<br>R: GGTTAGCCATGAAGGTTCCA	145

8	*AhGolS4*	Galactinol Synthase (arahy.KNQB38)	F: TTGGGAGTTTGTGGAGTATAGC<br>R: CCTTAGGCCATTGAACCTTATCA	203
9	*AhGolS6*	Galactinol Synthase (arahy.T7DX97)	F: TGCCTTCGTGACTTTCCTTG<br>R: GATGTTGCGGTGTTCTTGAG	142
10	*AhRS14*	Raffinose Synthase (arahy.1YYJ7Z)	F: GTGTACATGGTGAAAGACCAC<br>R: GGATTGAATTGAGCCTCCAGA	186
11	*AhRS4*	Raffinose Synthase (arahy.WB441U)	F: AGTGTACATGGTGAAAAACCAA<br>R: GGATTGAATTGAGCCTCCAGA	187
12	*AhRS6*	Raffinose Synthase (arahy.82P426)	F: GATGGTGTAAGGTAGAGAAGAGG<br>R: TATCACCGGAACTGAAGCAC	194
13	*AhRSV*	Raffinose Synthase (arahy.AY7U99)	F: TCAACTCACAGGGCCTATTTC<br>R: CACCTGAAAGCTGGTTCTTATTG	104
14	*AhRS8*	Raffinose Synthase (arahy.PF1JUH)	F: GAACATGCTCAACTCTGGC<br>R: TCACACCCAACCCATTAATCT	149
15	*AhRS3*	Raffinose Synthase (arahy.SCX26U)	F: TGAACATGCTTAACTCCGGT<br>R: CCACATCCACCCCATTAATCT	150
16	*AhSS7*	Stachyose Synthase (arahy.T832D6)	F: TGGGATCCTAAAGAACACACG<br>R: ACTGCCACAAAGCTGAAGA	242

Table S3. Ranking of genes according to their stability value or standard deviation by integration of different ranking methods

Genes	Comprehensive Ranking	Delta Ct	NormFinder	GeNorm	BestKeeper	Overall Ranking
	Geomean of ranking values	Average of SD	Stability value	Stability value	SD	
AhUBI1	1.41	1.26	0.128	0.256	0.87	1
AhADH3	1.78	1.30	0.128	0.256	0.89	2
AhActin1	2.28	1.34	0.378	0.428	0.83	3
AhG6PD	3.94	1.57	0.963	0.703	0.89	4
AhUBC1	4.47	1.61	0.868	0.883	0.89	5
AhActin11	6.24	2.54	2.316	1.350	2.25	6
AhELF1B	6.74	2.87	2.693	1.784	2.17	7

Different ranking tools were integratively used in RefFinder software to select the best reference gene as endogenous control. *Ah* stands for *Arachis hypogaea*, UBI1 = Polyubiquitin, ADH3 = Alcohol dehydrogenase class III, ELF1B = Elongation factor1-beta, G6PD = Glucose 6-phosphatedehydrogenase, UBC1 = Ubiquitin C, SD = Standard Deviation.

Table S4. Distribution of genes involved in biosynthesis of Raffinose Family Oligosaccharides in *Arachis hypogaea* chromosome and size of each chromosome.

Family	Gene Name	Gene ID		Chromosome	Start (bp)	End (bp)
		Peanut Genome Resources	PeanutBase			
Galactinol Synthase	*AhGolS1*	AH06G04780	arahy.N6N04K	A6	7372956	7374154
	AhGolS2	AH09G11340	arahy.9HBV2U	A9	19359461	19361880
	AhGolS3	AH09G11360	arahy.FK4NNE	A9	19369230	19373832

	AhGolS4	AH09G12530	arahy.KNQB38	A9	23295792	23297552
	AhGolS5	AH16G08210	arahy.HZ3A81	B6	13919203	13921118
	AhGolS6	AH16G09600	arahy.T7DX97	B6	15938464	15940252
	AhGolS7	AH19G14790	arahy.96F7ZV	B9	24050668	24052744
	AhGolS8	AH19G14820	arahy.96F7ZV	B9	24062000	24065052
	AhGolS9	AH19G16350	arahy.B1GHBY	B9	29335476	29337107
**Raffinose Synthase**	*AhRS1*	AH01G25000	arahy.UR837P	A1	97414835	97417909
	AhRS2	AH03G22950	arahy.7F6VM6	A3	43550202	43555938
	AhRS3	AH03G23470	arahy.SCX26U	A3	44115868	44122524
	AhRS4	AH03G23920	arahy.WB441U	A3	46419360	46431663
	AhRS5	AH05G24120	arahy.B3XDAM	A5	91409141	91415144
	AhRS6	AH06G01900	arahy.82P426	A6	3781168	3785408
	AhRS7	AH07G00130	arahy.SCX26U	A7	363064	365523
	AhRS8	AH07G00140	arahy.PF1JUH	A7	365572	368992
	AhRS9	AH07G01370	arahy.C3FF4U	A7	1289070	1292937
	AhRS10	AH07G17720	No hits	A7	51980576	51981067
	AhRS11	AH09G16320	arahy.UR837P	A9	53685525	53685821
	AhRS12	AH11G32960	arahy.SM3TC1	B1	145745289	145748844
	AhRS13	AH13G01440	arahy.4X3ZEJ	B3	1834889	1840567
	AhRS14	AH13G26910	arahy.1YYJ7Z	B3	48501704	48514269
	AhRS15	AH15G31330	arahy.DBZB80	B5	145581591	145586985
	AhRS16	AH16G04370	arahy.3HRB74	B6	8188588	8192653
	AhRS17	AH17G01490	arahy.C3FF4U	B7	1941587	1945308
**Stachyose Synthase**	*AhSS1*	AH01G31080	arahy.T832D6	A1	105403482	105403784
	AhSS2	AH06G04600	arahy.RWK4UY	A6	7153076	7156467
	AhSS3	AH06G04610	arahy.3E1AF2	A6	7161436	7164458
	AhSS4	AH07G07050	arahy.3E1AF2	A7	7460657	7460887
	AhSS5	AH13G25550	arahy.TR51Z4	B3	42936278	42937422
	AhSS6	AH15G16580	arahy.Y2LGU1	B5	74218184	74218342
	AhSS7	AH16G08010	arahy.T832D6	B6	13618714	13622241
	AhSS8	AH16G08020	arahy.Y2LGU1	B6	13636266	13639347

**Chromosome**	**A1**	**A2**	**A3**	**A4**	**A5**	**A6**	**A7**	**A8**	**A9**	**A10**
**Length (Mb)**	111.624	99.792	143.207	122.065	116.167	109.265	83.117	51.955	121.059	114.520
**Chromosome**	**B1**	**B2**	**B3**	**B4**	**B5**	**B6**	**B7**	**B8**	**B9**	**B10**
**Length (Mb)**	149.380	122.214	150.766	135.086	153.119	153.789	132.999	136.777	159.155	140.659

Table S5. Sugar profile and content of RFOs (g 100 g^{-1}) in the dry seeds of peanut

Genotypes	Agronomic Group	Inositol (g 100 g^{-1})	Glucose (g 100 g^{-1})	Sucrose (g 100 g^{-1})	Raffinose (g 100 g^{-1})	Stachyose (g 100 g^{-1})	Total RFOs (g 100 g^{-1})
GG 20	VB	0.06 cd	0.003 b	5.27 e	0.06 b	0.49 b	0.55 bc
GG 5	SB	0.08 ab	0.006 a	7.08 a	0.09 a	0.43 c	0.52 c
GG 7	SB	0.09 a	0.001 b	6.32 bc	0.06 b	0.34 d	0.40 d
Girnar 2	VB	0.04 e	0.001 b	6.26 c	0.07 ab	0.55 ab	0.62 ab
Girnar 3	SB	0.04 de	0.008 a	6.06 d	0.05 b	0.52 b	0.58 bc
TG37A	SB	0.07 bc	0.001 b	6.36 b	0.07 ab	0.60 a	0.67 a
C.V. (%)		15.48	32.61	0.82	16.65	6.60	7.14
SEm±		0.01	0.001	0.01	0.01	0.02	0.02
S.E.D.		0.01	0.001	0.04	0.01	0.03	0.03
LSD (p<0.05)		0.02	0.002	0.09	0.02	0.06	0.07
ProbF		0.000**	0.000 **	0.000 **	0.028 *	0.000 **	0.000 **

All values are mean of three replications. Mean values with different letters within the same column are statistically different (p-value < 0.05). VB = Virginia Bunch, SB = Spanish Bunch C.V. (%) = Coefficient of Variation, S.E.M = Standard Error of the Mean, S.E.D. = Standard Error of Difference, LSD = Least Significant Difference, ProbF = Probability from an *F* distribution (* denotes significant and ** denotes highly significant).

Table S6. Glucose, Sucrose and RFOs content (g 100 g^{-1}) in the maturing stage (S1) and matured stage (S2) of seeds in contrast peanut genotypes.

Variety	Seed Stage	D-Glucose (g 100g^{-1})	Sucrose (g 100g^{-1})	Total RFOs (g 100g^{-1})
TG37A	S1	0.05 ± 0.002	10.23 ± 0.023	0.54 ± 0.023
	S2	0.04 ± 0.002	6.09 ± 0.030	0.72 ± 0.011
GG7	S1	0.06 ± 0.002	9.55 ± 0.030	0.26 ± 0.013
	S2	0.06 ± 0.001	6.09 ± 0.011	0.47 ± 0.013
Girnar 2	S1	0.12 ± 0.013	10.23 ± 0.026	0.49 ± 0.016
	S2	0.09 ± 0.003	6.34 ± 0.027	0.64 ± 0.018
C.V. (%)		14.39	0.54	5.42
SEm±		0.01	0.03	0.02
S.E.D.		0.01	0.04	0.02
LSD (p<0.05)		0.02	0.08	0.05
ProbF		0.000 **	0.000 **	0.000 **

All values are mean of three replications ± Standard Error. S1 = maturing seed stage, S2 = matured seed stage, C.V. (%) = Coefficient of Variation, S.E.M = Standard Error of the Mean, S.E.D. = Standard Error of Difference, LSD = Least Significant Difference, ProbF = Probability from an *F* distribution (denotes highly significant).**

Table S7. Number of differentially expressed genes.

Condition	Significantly Expressed Genes	Up Regulated Genes	Down Regulated genes
GG7_S2 vs GG7_S1	2,321	622	1699
TG37A_S1 vs GG7_S1	6,341	2,690	3,651
TG37A_S2 vs GG7_S2	8,149	2,951	5,198
Girnar2_S2 vs Girnar2_S1	2,629	416	2,213
TG37A_S1 vs Girnar2_S1	5,015	1,775	3,240

TG37A_S2 vs Girnar2_S2	4,461	1,379	3,082
TG37A_S2 vs TG37A_S1	2,247	301	1,946

Seed maturity stages are denoted by S1 (maturing stage) and S2 (matured stage). Total number of tested genes were 62,987. The data is the average of three biological replicates.

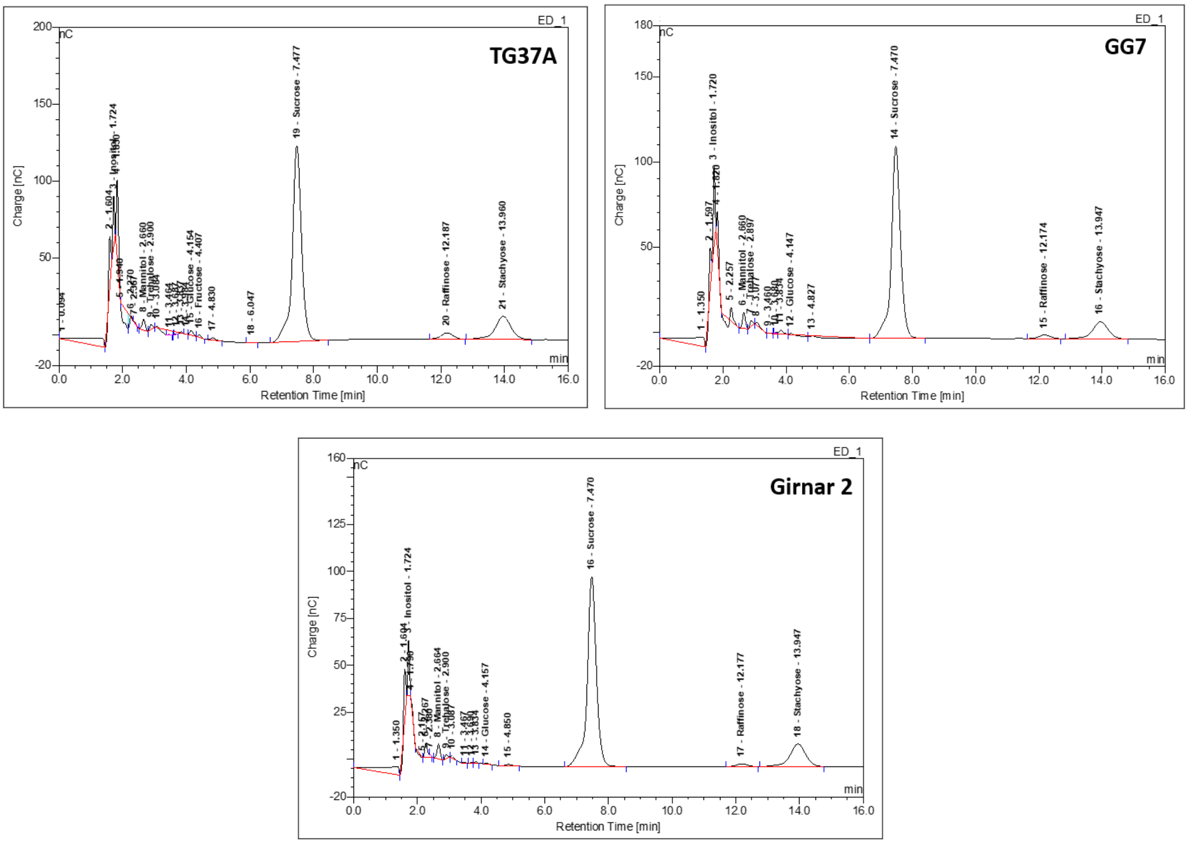

Fig. S1. Ion Chromatogram of peanut seed samples of contrast genotypes. Each peak represents the detected compounds and the values indicate the retention time. Data integration was done using Chormeleon software.

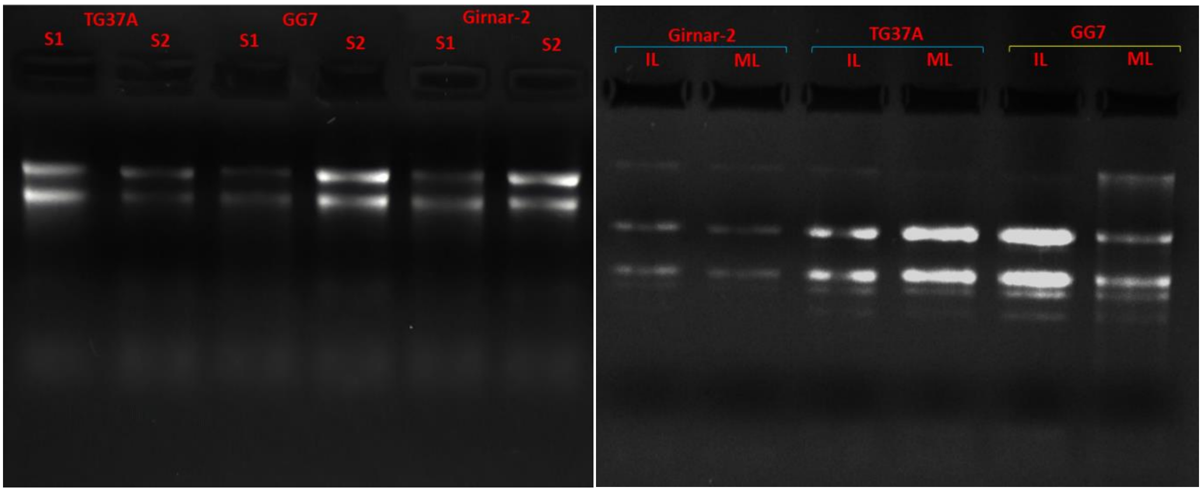

Fig. S2. Qualitative assessment of total RNA. The eluted RNA samples were run on 1.3% denaturing agarose gel electrophoresis and 28S, 18S, and 5S rRNA bands were separated, revealing the integrity of the RNA. **S1** = maturing seed, **S2** = matured seed, **IL** = immature leaf, **ML** = mature leaf.

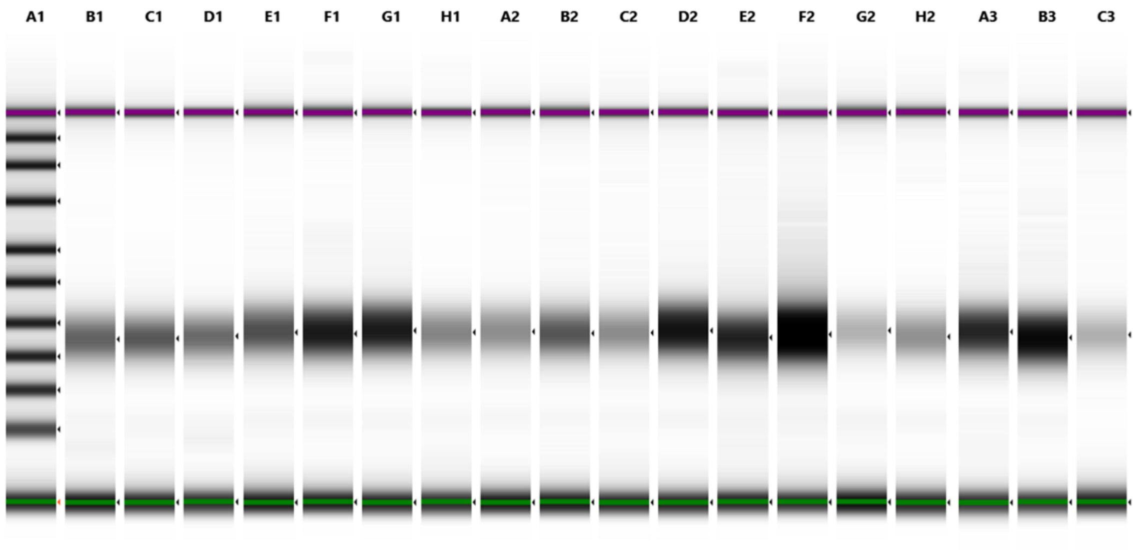

Fig. S3. Gel profile of cDNA libraries. cDNA libraries of three peanut genotypes at two seed stages qualified the criteria based on the presence of a broad peak in the range of 300bp to 1000bp, with an average size of 400 – 600 bp in the Agilent 4150 TapeStation system **A1** = Ladder, **B1** to **C3** are the sample wells.

(a) (b)

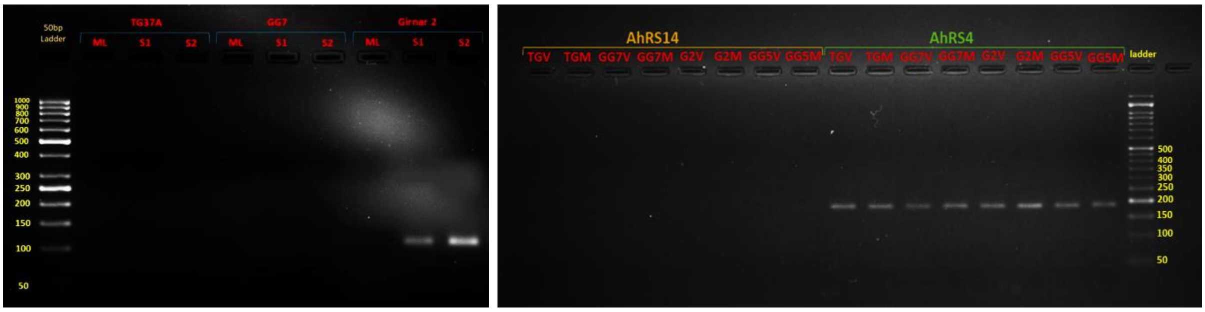

Fig. S4. Agarose gel electrophoresis images of different genes across peanut tissue samples. (a) *AY7U99* or *AhRSV* primer band pattern in Matured Leaf (ML), Maturing (S1) seed, Matured (S2) seed stage of Spanish genotypes (TG37A and GG7) and Virginia genotype (Girnar 2). Spanish gentotypes do not show any expression while Virginia genotype shows seed-specific expression when run on 3% denaturing agarose gel. **(b)** *AhRS4* and *AhRS14* gene present on chromosome A3/B3 shows a differential expression pattern. *AhRS*4 shows expression in the leaf while *AhRS14* does not. Leaf samples at two different stages, vegetative (V) and mature (M) in four peanut genotypes - TG37A (TG), GG7, Girnar 2 (G2), and GG5 are shown here. Samples were run on 3% denaturing agarose gel. A 50bp DNA ladder was used.

TG37A vs GG7 (S1)				
Gene	$\Delta\Delta C_T$	Fold Change ($2^{-\Delta\Delta C_T}$)	log 2 Fold Change	
			qPCR	RNA-Seq
AhGolS4	-2.065	4.185	2.065	2.848
AhGolS6	-6.015	64.657	6.015	3.801
AhRS14	-7.257	152.973	7.257	6.340
AhRS4	-2.114	4.329	2.114	7.165
AhRS6	-0.648	1.567	0.648	1.168
AY7U99	-2.546	5.840	2.546	0.000
AhRS8	-3.699	12.984	3.699	1.467
AhRS3	-5.220	37.269	5.220	3.087
AhSS7	-3.834	14.264	3.834	0.803

A

TG37A vs GG7 (S2)				
Gene	$\Delta\Delta C_T$	Fold Change ($2^{-\Delta\Delta C_T}$)	log 2 Fold Change	
			qPCR	RNA-Seq
AhGolS4	-0.251	1.190	0.251	-0.048
AhGolS6	-1.432	2.699	1.432	0.488
AhRS14	-7.341	162.078	7.341	4.575
AhRS4	-4.217	18.592	4.217	6.229
AhRS6	0.976	0.508	-0.976	-0.918
AY7U99	-3.577	11.930	3.577	1.896
AhRS8	-1.743	3.347	1.743	-3.546
AhRS3	-1.180	2.266	1.180	-2.524
AhSS7	0.888	0.540	-0.888	-2.187

B

TG37A vs Girnar2 (S1)				
Gene	$\Delta\Delta C_T$	Fold Change ($2^{-\Delta\Delta C_T}$)	log 2 Fold Change	
			qPCR	RNA-Seq
AhGolS4	-8.702	416.556	8.702	6.021
AhGolS6	-3.185	9.093	3.185	2.211
AhRS14	-1.758	3.383	1.758	5.897
AhRS4	0.680	0.624	-0.680	5.909
AhRS6	-7.009	128.816	7.009	7.482
AY7U99	5.961	0.016	-5.961	-10.466
AhRS8	0.242	0.845	-0.242	-0.341
AhRS3	0.162	0.894	-0.162	-0.067
AhSS7	1.082	0.472	-1.082	-0.101

C

TG37A vs Girnar2 (S2)				
Gene	$\Delta\Delta C_T$	Fold Change ($2^{-\Delta\Delta C_T}$)	log 2 Fold Change	
			qPCR	RNA-Seq
AhGolS4	-2.954	7.748	2.954	1.582
AhGolS6	0.254	0.839	-0.254	-0.997
AhRS14	1.684	0.311	-1.684	0.397
AhRS4	2.440	0.184	-2.440	0.501
AhRS6	-6.703	104.187	6.703	5.969
AY7U99	8.657	0.002	-8.657	-8.981
AhRS8	0.813	0.569	-0.813	-4.072
AhRS3	2.101	0.233	-2.101	-3.444
AhSS7	3.494	0.089	-3.494	-2.563

D

Girnar2 vs GG7 (S1)				
Gene	$\Delta\Delta C_T$	Fold Change ($2^{-\Delta\Delta C_T}$)	log 2 Fold Change	
			qPCR	RNA-Seq
AhGolS4	6.637	0.010	-6.637	-3.176
AhGolS6	-2.830	7.111	2.830	1.594
AhRS14	-5.499	45.219	5.499	0.585
AhRS4	-2.794	6.937	2.794	1.585
AhRS6	6.361	0.012	-6.361	-6.316
AY7U99	-8.507	363.742	8.507	7.462
AhRS8	-3.941	15.357	3.941	1.825
AhRS3	-5.382	41.701	5.382	3.156
AhSS7	-4.917	30.202	4.917	0.905

E

Girnar2 vs GG7 (S2)				
Gene	$\Delta\Delta C_T$	Fold Change ($2^{-\Delta\Delta C_T}$)	log 2 Fold Change	
			qPCR	RNA-Seq
AhGolS4	2.703	0.154	-2.703	-1.634
AhGolS6	-1.686	3.217	1.686	1.485
AhRS14	-9.025	520.779	9.025	4.184
AhRS4	-6.657	100.915	6.657	5.769
AhRS6	7.679	0.005	-7.679	-6.888
AY7U99	-12.234	4816.121	12.234	7.831
AhRS8	-2.555	5.878	2.555	0.531
AhRS3	-3.282	9.725	3.282	0.920
AhSS7	-2.606	6.090	2.606	0.376

F

TG37A (S2 vs S1)				
Gene	$\Delta\Delta C_T$	Fold Change ($2^{-\Delta\Delta C_T}$)	log 2 Fold Change	
			qPCR	RNA-Seq
AhGolS4	4.429	0.046	-4.429	-4.225
AhGolS6	1.232	0.426	-1.232	-0.591
AhRS14	-2.552	5.863	2.552	1.375
AhRS4	-3.060	8.341	3.060	1.399
AhRS6	-0.070	1.050	0.070	-0.547
AY7U99	0.430	0.742	-0.430	1.896
AhRS8	-1.517	2.862	1.517	-2.674
AhRS3	0.548	0.684	-0.548	-2.608
AhSS7	2.383	0.192	-2.383	-1.792

G

TG37A (Leaf vs Seed)			
Gene	$\Delta\Delta C_T$	Fold Change ($2^{-\Delta\Delta C_T}$)	log 2 Fold Change
AhGolS4	2.880	0.136	-2.880
AhGolS6	5.596	0.021	-5.596
AhRS14	7.312	0.006	-7.312
AhRS4	-1.257	2.390	1.257
AhRS6	14.025	0.000	-14.025
AY7U99	0.548	0.684	-0.548
AhRS8	2.220	0.215	-2.220
AhRS3	2.217	0.215	-2.217
AhSS7	3.566	0.084	-3.566

H

GG7 (S2 vs S1)				
Gene	$\Delta\Delta C_T$	Fold Change ($2^{-\Delta\Delta C_T}$)	log 2 Fold Change	
			qPCR	RNA-Seq
AhGolS4	2.614	0.163	-2.614	-1.329
AhGolS6	-3.350	10.198	3.350	2.722
AhRS14	-2.468	5.533	2.468	3.140
AhRS4	-0.958	1.942	0.958	2.336
AhRS6	-1.694	3.236	1.694	1.540
AY7U99	1.460	0.363	-1.460	0.000
AhRS8	-3.473	11.101	3.473	2.339
AhRS3	-3.491	11.244	3.491	3.004
AhSS7	-2.339	5.060	2.339	1.199

I

GG7 (Leaf vs Seed)			
Gene	$\Delta\Delta C_T$	Fold Change ($2^{-\Delta\Delta C_T}$)	log 2 Fold Change
AhGolS4	2.152	0.225	-2.152
AhGolS6	4.949	0.032	-4.949
AhRS14	2.243	0.211	-2.243
AhRS4	-2.165	4.485	2.165
AhRS6	14.878	0.000	-14.878
AY7U99	-5.225	37.409	5.225
AhRS8	0.293	0.816	-0.293
AhRS3	0.723	0.606	-0.723
AhSS7	10.517	0.001	-10.517

J

Girnar2 (S2 vs S1)				
Gene	$\Delta\Delta C_T$	Fold Change ($2^{-\Delta\Delta C_T}$)	log 2 Fold Change	
			qPCR	RNA-Seq
AhGolS4	-1.320	2.496	1.320	0.214
AhGolS6	-2.206	4.614	2.206	2.616
AhRS14	-5.994	63.726	5.994	6.876
AhRS4	-4.820	28.254	4.820	6.807
AhRS6	-0.377	1.298	0.377	0.966
AY7U99	-2.267	4.812	2.267	0.410
AhRS8	-2.087	4.249	2.087	1.058
AhRS3	-1.391	2.622	1.391	0.769
AhSS7	-0.029	1.020	0.029	0.670

K

Girnar2 (Leaf vs Seed)			
Gene	$\Delta\Delta C_T$	Fold Change ($2^{-\Delta\Delta C_T}$)	log 2 Fold Change
AhGolS4	0.500	0.707	-0.500
AhGolS6	6.724	0.009	-6.724
AhRS14	11.710	0.000	-11.710
AhRS4	2.583	0.167	-2.583
AhRS6	6.946	0.008	-6.946
AY7U99	8.415	0.003	-8.415
AhRS8	3.003	0.125	-3.003
AhRS3	4.230	0.053	-4.230
AhSS7	9.385	0.001	-9.385

L

Fig. S5. Analysis of peanut tissue samples under different combinations of contrast genotype for determination of expression. A. Maturing seed stage (S1) of High RFOs variety (TG37A) vs Low RFOs variety (GG7), **B**. Matured seed stage (S2) of High RFOs variety (TG37A) vs Low RFOs variety (GG7), **C**. High RFOs variety of Spanish group (TG37A) vs Virginia group (Girnar 2) at maturing seed (S1) stage, **D**. High RFOs variety of Spanish group (TG37A) vs Virginia group (Girnar 2) at matured seed (S2) stage, **E**. High RFOs Virginia variety (Girnar 2) vs Low RFOs Spanish variety (GG7) at maturing seed (S1) stage, **F**. High RFOs Virginia variety (Girnar 2) vs Low RFOs Spanish variety (GG7) at matured seed (S2) stage, **G**. High RFO cultivar (TG37A) matured seed (S2) vs maturing seed (S1) stage, **H.** High RFO Spanish cultivar (TG37A) leaf vs seed, **I.** low RFO cultivar (GG7) matured seed (S2) vs maturing seed (S1) stage, **J.** Low RFO Spanish cultivar (GG7) leaf vs seed, **K.** High RFO Virginia Cultivar (Girnar 2) matured seed (S2) vs maturing seed (S1) stage, **L.** High RFO Virginia Cultivar (Girnar 2) leaf vs seed.

(a)

(b)

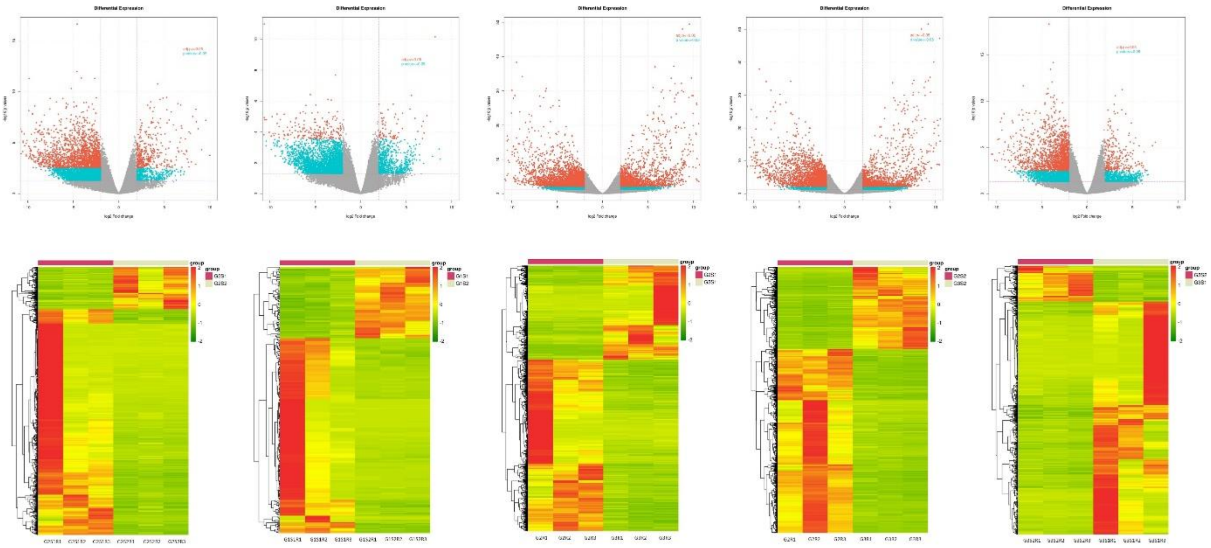

Fig. S6. Expression profile of the significant differentially expressed genes across the contrast genotypes. (a) Volcano plot showing differential expression profile of genes. Blue indicates log2 fold change ≥ 2 and p value ≤ 0.05. “Red” dots indicate absolute log2 fold change ≥ 2 and FDR/adjusted p value ≤ 0.05. From left to right, Girnar 2 (S2 vs S1), GG7 (S2 vs S1), Girnar 2 vs TG37A (S1), Girnar 2 vs TG37A (S2), TG37A (S2 vs S1). **(b)** Heatmap showing the DEGs in Girnar 2 (S2 vs S1), GG7 (S2 vs S1), Girnar 2 vs TG37A (S1), Girnar 2 vs TG37A (S2), TG37A (S2 vs S1) (*left to right*). R1, R2, R3 represent the three replications.
